# Comparing the Efficacy and Safety Profile of Triple Fixed-Dose Combinations in COPD: A Meta-Analysis and IBiS Score

**DOI:** 10.3390/jcm11154491

**Published:** 2022-08-01

**Authors:** Paola Rogliani, Josuel Ora, Francesco Cavalli, Mario Cazzola, Luigino Calzetta

**Affiliations:** 1 Unit of Respiratory Medicine, Department of Experimental Medicine, University of Rome “Tor Vergata”, 00133 Rome, Italy; paola.rogliani@uniroma2.it (P.R.); josuel78@gmail.com (J.O.); mario.cazzola@uniroma2.it (M.C.); 2 Division of Respiratory Medicine, University Hospital Policlinico Tor Vergata, 00133 Rome, Italy; frn.cavalli@gmail.com; 3 Respiratory Disease and Lung Function Unit, Department of Medicine and Surgery, University of Parma, 43126 Parma, Italy

**Keywords:** efficacy, COPD, indirect comparison, network meta-analysis, rank, safety, triple FDCs

## Abstract

Background: Triple fixed-dose combination (FDC) therapy is recommended in severe chronic obstructive pulmonary disease (COPD) patients experiencing frequent exacerbations and/or symptoms not controlled by dual FDCs. Since no randomized controlled trials (RCTs) have directly compared the different inhaled corticosteroid/long-acting β_2_-adrenoceptor agonist/long-acting muscarinic antagonist (ICS/LABA/LAMA) FDCs, we performed a meta-analysis to compare the impact of the current available ICS/LABA/LAMA FDCs in COPD. Methods: A meta-analysis was performed by connecting beclomethasone dipropionate/formoterol fumarate/glycopyrronium bromide or glycopyrrolate (BDP/FOR/GLY), budesonide (BUD)/GLY/FOR, and fluticasone furoate/umeclidinium bromide/vilanterol (FF/UMEC/VI) FDCs via ICS/LABA or LABA/LAMA FDCs arms. The safety and efficacy profiles were investigated, and the Implemented Bidimensional Surface under the cumulative ranking curve analysis (IBiS) was carried out. Protocol registration: CRD42022301189. Results: Data from 21,809 COPD patients were extracted from the ETHOS, IMPACT, KRONOS, and TRILOGY studies. No significant (*p* > 0.05) differences were detected across the triple FDCs with respect to the risk of exacerbation, trough forced expiratory volume in the first second (FEV_1_), transition dyspnea index (TDI), St. George’s Respiratory Questionnaire (SGRQ), risk of serious adverse events (SAEs), cardiovascular (CV) SAEs, pneumonia, and all-cause mortality. According to IBiS score, BDP/FOR/GLY 200/12/25 µg twice daily (BID) was the FDC reporting the best combined efficacy/safety profile (area 41.41%), although FF/UMEC/VI 100/62.5/25 µg once daily (QD) showed the greatest efficacy profile (50.54%). The protection against mortality related to the dose of ICS. Conclusions: All triple FDCs are effective and safe in COPD regardless of the regimen of administration (twice daily vs. once daily), with no relevant difference in the risk of CV SAEs and pneumonia.

## 1. Introduction

Combining an inhaled corticosteroid (ICS) with a long-acting β_2_-adrenoceptor agonist (LABA) and a long-acting muscarinic antagonist (LAMA) in a fixed-dose combination (FDC) is a keystone in the treatment of severe forms of chronic obstructive pulmonary disease (COPD) [1]. Nevertheless, recognizing that COPD is a complex and heterogeneous disorder [2], to date, there has been a debate if ICS/LABA/LAMA FDCs may represent a real precision medicine opportunity or if such a therapeutic approach may lead to a simplistic interpretation of symptoms and risk of acute exacerbation of COPD (AECOPD), thus promoting the typical “one size fit all” attitude [3].

The current triple FDCs for the treatment of COPD include different ICS (i.e., beclomethasone dipropionate (BDP), budesonide (BUD), or fluticasone furoate (FF)), LABA (i.e., formoterol fumarate (FOR) or vilanterol (VI)), and LAMA (i.e., glycopyrronium bromide or glycopyrrolate (GLY) or umeclidinium bromide (UMEC)) in the same formulation [4]. Looking at the specific pharmacokinetic (PK) and pharmacodynamic (PD) profiles of these mono-components, both bronchodilators and ICS [5,6,7,8], it is expected that the clinical impact in terms of efficacy and safety profile of each triple FDC may be modulated by the pharmacological characteristics of the molecules combined in the marketed formulations approved by the European Medicine Agency [9,10,11] and US Food and Drug Administration [12,13]. In this respect, although several large randomized controlled trials (RCTs) have demonstrated the superiority of triple FDCs vs. dual FDCs [14,15,16], to the best of our knowledge, no studies have directly compared different ICS/LABA/LAMA FDCs each other’s in COPD patients.

Moving from the hypothesis proposed by the physicist Richard Feynman that “the statements of science are not of what is true and what is not true, but statements of what is known with different degrees of certainty”, the aim of this meta-analysis was to perform a comparison across ICS/LABA/LAMA FDCs by providing ranks of efficacy and safety in COPD patients according to the level of evidence available from the current literature. To provide the greater degree of certainty, we previously performed a pairwise meta-analysis to identify the treatment comparison that could have introduced bias in the effect estimates, and then performed a Bayesian network leading to the Implemented Bidimensional Surface under the cumulative ranking curve analysis (IBiS), a score that ranks the probability that each intervention arm is the best in terms of efficacy and safety profile [17]. The iteration across the different ICS/LABA/LAMA FDCs passed through the ICS/LABA and LABA/LAMA FDCs nodes according to the molecules included in the combinations and inhaler devices to prevent the risk of bias across studies and maximize the quality of evidence [4,18].

## 2. Materials and Methods

### 2.1. Search Strategy and Study Eligibility

**Figure 1:**
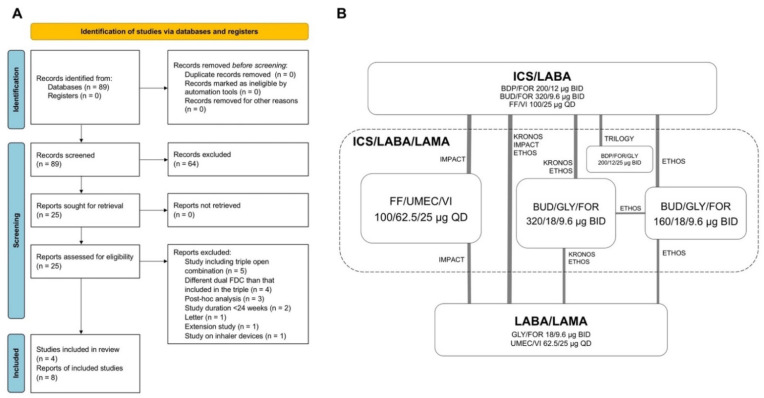
PRISMA 2020 flow diagram (**A**) and diagram displaying the Bayesian network across the treatments (**B**). The links between the nodes indicate the direct comparisons between pairs of treatments; the thickness of lines is proportional to the number of patients comparing pairs of treatment head-to-head, and the area of the boxes is proportional to the number of patients receiving the same treatment. BDP: beclomethasone dipropionate; BID: bis in die, twice daily; BUD: budesonide; FDC: fixed-dose combination; FF, fluticasone furoate; FOR: formoterol fumarate; GLY: glycopyrronium bromide or glycopyrrolate; ICS: inhaled corticosteroid; LABA: long-acting β_2_-adrenoceptor agonist; LAMA: long-acting muscarinic antagonist; PRISMA: Preferred Reporting Items for Systematic Reviews and Meta-Analyses; QD: quaque die, once daily; UMEC: umeclidinium bromide; VI: vilanterol.

This quantitative synthesis was registered to the international prospective register of systematic reviews (PROSPERO ID: CRD42022301189) and performed in agreement with the Preferred Reporting Items for Systematic Reviews and Meta-Analyses Protocols (PRISMA-P) [19]. The relative flow diagram and the Bayesian network with nodes are shown in Figure 1A,B. This study satisfied all the recommended items reported by the PRISMA-P checklist (Table S1) [19].

A comprehensive literature search was performed for RCTs evaluating the efficacy and safety of triple FDC therapies for the treatment of COPD. The PICO (Patient Problem, Intervention, Comparison, and Outcome) [20] framework was applied as follows: the “patient problem” was COPD, the “intervention” regarded triple ICS/LABA/LAMA FDCs, the “comparison” was performed across the interventions, and the “outcomes” were the risk of moderate or severe AECOPD, lung function, dyspnea, quality of life, risk of total serious adverse events (SAEs), cardiovascular (CV) SAEs, pneumonia, and all-cause mortality.

The search was performed in ClinicalTrials.gov, Cochrane Central Register of Controlled Trials (CENTRAL), Embase, EU Clinical Trials Register, MEDLINE, Scopus, and Web of Science, in order to provide relevant studies published up to 6 September 2021 (detailed information available in the Supplementary Materials). Citations of previous published reviews were checked to select further pertinent RCTs, if any [21]. Literature search results were uploaded to Eppi-Reviewer 4 (EPPI-Centre Software, London, UK; detailed information available in the Supplementary Materials).

### 2.2. Study Selection

RCTs that enrolled COPD patients, lasting ≥24 weeks, and that included at least one arm assessing the impact of triple ICS/LABA/LAMA FDCs compared to the same dual FDC (either ICS/LABA or LABA/LAMA) as in the triple FDC and were administered via the same device were included in the quantitative analysis. Two reviewers independently examined the studies, and any difference in opinion concerning the selection of relevant RCTs from literature searches and databases was resolved by consensus.

### 2.3. Data Extraction

Data were extracted from published papers, Supplementary Materials, and the public database ClinicalTrials.gov.

Data were checked for study characteristics and duration, number of analyzed patients, treatments with doses of medications and regimen of administration, main inclusion criteria, history, rate, and frequency of AECOPD, age, gender, smoking habit, forced expiratory volume in the first second (FEV_1_), level of FEV_1_ reversibility, blood eosinophil count at baseline, blood eosinophil count, Jadad score [22], and Cochrane risk of bias [23].

Data were extracted in agreement with Data Extraction for Complex Meta-Analysis (DECiMAL) recommendations [24], and the inter- and intra-rater reliability for data abstraction was assessed via Cohen’s kappa score (detailed information available in the Supplementary Materials) [25].

### 2.4. Endpoints

The efficacy endpoints of this meta-analysis were the risk of moderate or severe AECOPD, the change from baseline in trough FEV_1_, transition dyspnea index (TDI), and St. George’s Respiratory Questionnaire (SGRQ); the safety endpoints were the risk of total SAEs, CV SAEs, pneumonia, and all-cause mortality.

### 2.5. Data Synthesis and Analysis

Results of the pairwise meta-analysis comparing ICS/LABA/LAMA FDCs with dual FDCs were expressed as relative risk (RR) or mean difference (MD) and 95% confidence interval (95% CI), depending on the analyzed variables. Since data were selected from a series of studies performed by researchers operating independently and a common effect size cannot be assumed, a binary random-effects model was used in the pairwise meta-analysis to balance the study weights and adequately estimate the 95% CI of the mean distribution of drugs effect on the investigated variables. The test for heterogeneity (I^2^) was performed to quantify the between-study dissimilarity [26], and sensitivity analysis was carried out to identify the studies that introduced substantial level of heterogeneity (I^2^ > 50%) [27].

A network meta-analysis indirectly compared the efficacy and safety of the different triple ICS/LABA/LAMA FDCs (detailed information available in the Supplementary Materials). Results of the network meta-analysis are expressed as the relative effect (RE) and 95% credible interval (95% CrI).

A sensitivity analysis was performed by excluding from the Bayesian network the studies that introduced substantial heterogeneity as resulting from the pairwise meta-analysis. After that, the probability that each intervention arm was the most effective/safe was calculated by counting the proportion of iterations of the chain in which each intervention arm had the best relative effect, as well as the surface under the cumulative ranking curve analysis (SUCRA), representing the summary of these probabilities [28]. The SUCRA is 1 when a treatment is considered to be the best, and 0 when a treatment is considered to be the worst [29]. In this study, the ranks resulting from SUCRA were combined and plotted on different axes to produce radar charts, thus providing the IBiS score in which a larger percentage area covered by the radar chart indicates a greater efficacy and safety profile of each ICS/LABA/LAMA FDC [17].

### 2.6. Quality of Studies, Risk Bias, and Evidence Profile

The summary of the risk of bias for each included RCT was analyzed via the Jadad score [22] and Cochrane Risk of Bias 2 (RoB 2) [23] (detailed information available in the Supplementary Materials).

The risk of bias was checked via the normalized consistency/inconsistency analysis (detailed information available in the Supplementary Materials) [29].

The quality of the evidence was assessed via the Grading of Recommendations Assessment, Development, and Evaluation (GRADE) system (detailed information available in the Supplementary Materials) [23].

Two reviewers independently assessed the quality of studies, risk bias, and evidence profile, and any difference in opinion was resolved by consensus.

### 2.7. Software and Statistical Significance

The software used for the analysis is reported in the Supplementary Materials. The statistical significance of the effect estimates was assessed for *p* < 0.05.

## 3. Results

### 3.1. Study Characteristics

Data from 21,809 COPD patients were extracted from the ETHOS [14], IMPACT [15], KRONOS [30], and TRILOGY [16] phase III RCTs and from sub-studies of ETHOS [31] and IMPACT [32]. For the KRONOS [30] and TRILOGY [16] RCTs, data on all-cause mortality were extracted from primary publications, whereas those from the ETHOS [33] and IMPACT [34] studies were extracted from the final retrieved datasets. The relevant studies and patient characteristics are described in Table 1. All the RCTs [14,15,16,30] were characterized by medium to high quality according to Jadad score, and the definition of moderate and severe AECOPD was generally consistent across the studies (Table S2). The inter-rater reliability for data abstraction was excellent (detailed information available in the Supplementary Materials).

### 3.2. Pairwise Meta-Analysis

#### 3.2.1. Efficacy

**Figure 2:**
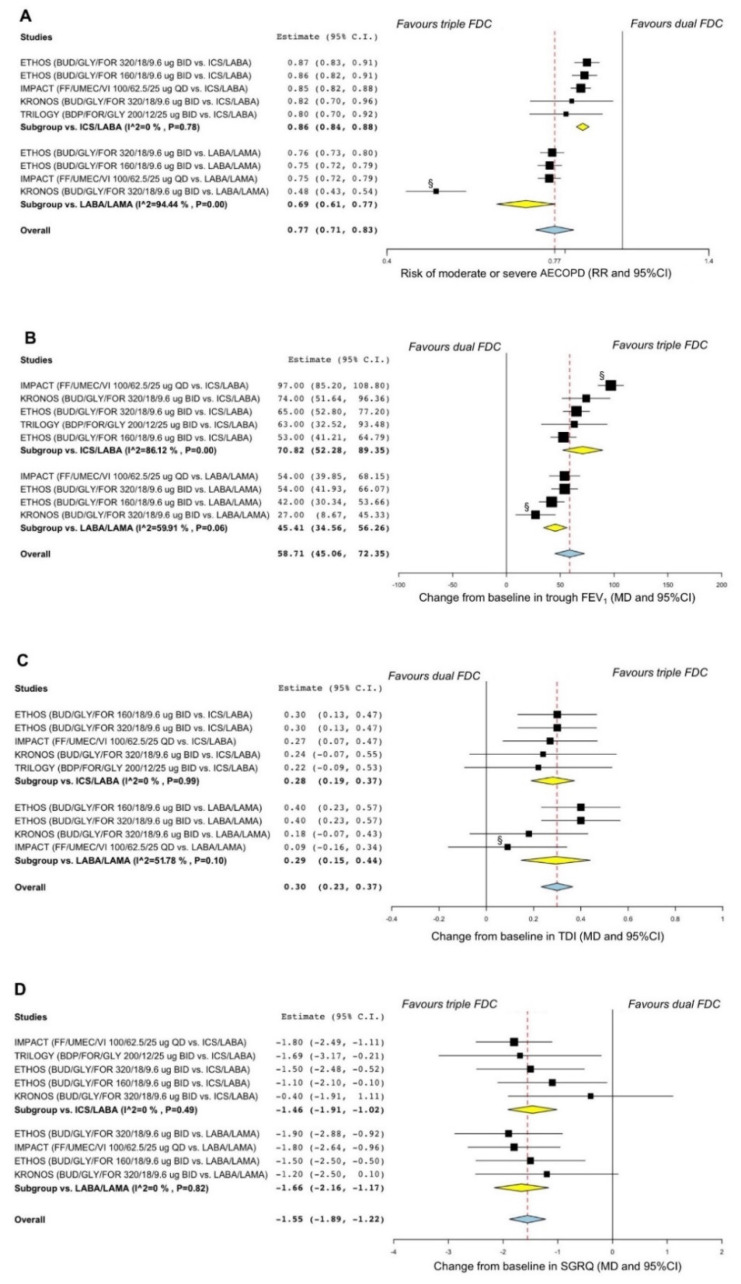
Forest plots of pairwise meta-analysis concerning the efficacy of triple ICS/LABA/LAMA FDCs vs. dual FDCs on the risk of moderate or severe AECOPD (**A**) and the change from baseline in trough FEV_1_ (**B**), TDI (**C**), and SGRQ (**D**). ^§^ Treatment comparison introducing substantial heterogeneity in the pairwise meta-analysis. AECOPD: acute exacerbation of COPD; BDP: beclomethasone dipropionate; BID: bis in die, twice daily; BUD: budesonide; COPD: chronic obstructive pulmonary disease; FDC: fixed-dose combination; FEV_1_: forced expiratory volume in the first second; FF: fluticasone furoate; FOR: formoterol fumarate; GLY: glycopyrronium bromide or glycopyrrolate; ICS: inhaled corticosteroid; LABA: long-acting β_2_-adrenoceptor agonist; LAMA: long-acting muscarinic antagonist; MD: mean difference; QD: quaque die, once daily; RR: relative risk; SGRQ: St. George’s respiratory questionnaire; TDI: transition dyspnea index; UMEC: umeclidinium bromide; VI: vilanterol.

When compared to dual FDCs, ICS/LABA/LAMA FDC significantly (*p* < 0.001) reduced the risk of moderate or severe AECOPD (overall RR 0.77, 95% CI 0.71–0.83). Moreover, ICS/LABA/LAMA FDC also significantly (*p* < 0.001) improved trough FEV_1_ (overall MD 59 mL, 95% CI 45–72), TDI (overall MD 0.30 points, 95% CI 0.23–0.37), and SGRQ (overall MD −1.55 points, 95% CI −1.89–−1.22) (Figure 2A–D).

According to the sensitivity analysis, the KRONOS [30] and IMPACT [34] RCTs introduced substantial heterogeneity on AECOPD, trough FEV_1_, and TDI. After resolving heterogeneity, the effect estimates resulting from the sensitivity analysis confirmed results obtained from the overall pairwise meta-analysis (Figure S1A–C).

#### 3.2.2. Safety

**Figure 3:**
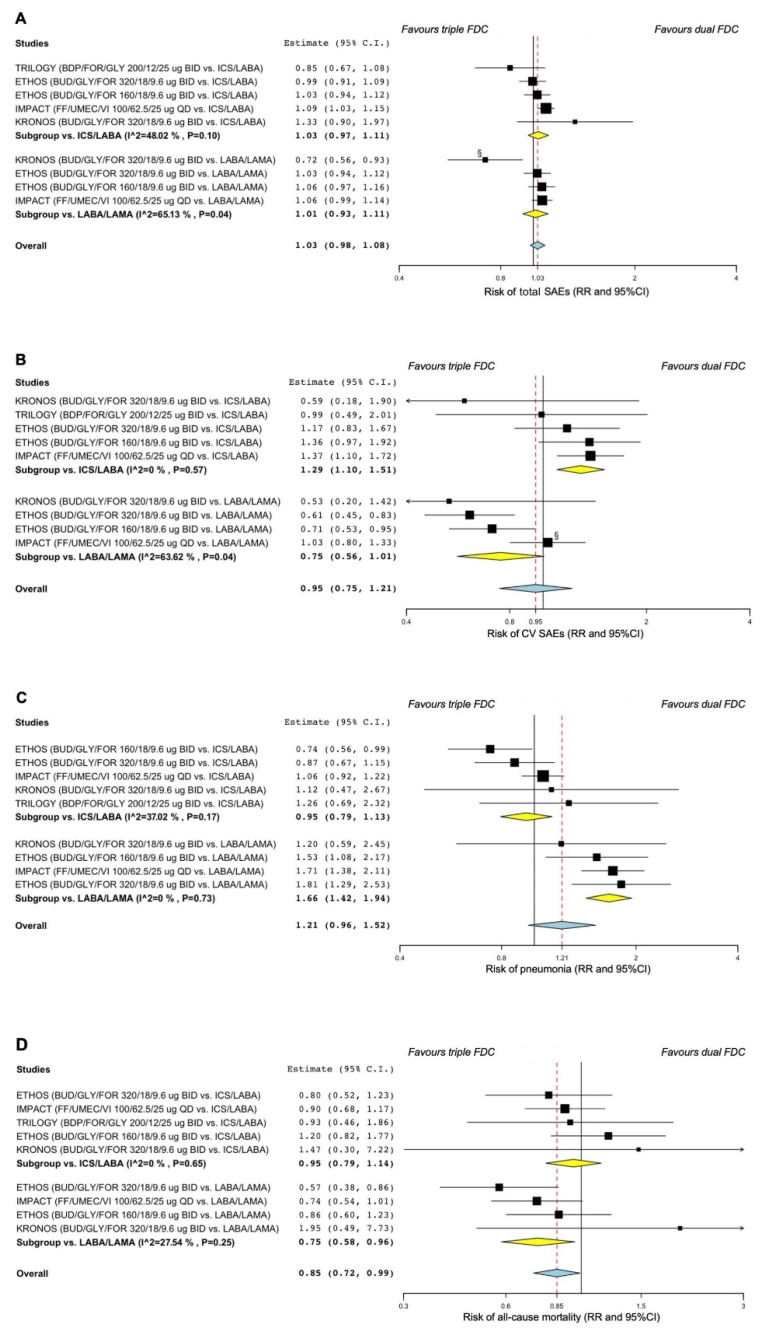
Forest plots of pairwise meta-analysis concerning the safety of triple ICS/LABA/LAMA FDCs vs. dual FDCs on the risk of total SAEs (**A**), CV SAEs (**B**), pneumonia (**C**), and all-cause mortality (**D**). ^§^ Treatment comparison introducing substantial heterogeneity in the pairwise meta-analysis. BDP: beclomethasone dipropionate; BID: bis in die, twice daily; BUD: budesonide; CV: cardiovascular; FDC: fixed-dose combination; FF: fluticasone furoate; FOR: formoterol fumarate; GLY: glycopyrronium bromide or glycopyrrolate; ICS: inhaled corticosteroid; LABA: long-acting β_2_-adrenoceptor agonist; LAMA: long-acting muscarinic antagonist; QD: quaque die, once daily; RR: relative risk; SAE: serious adverse event; UMEC: umeclidinium bromide; VI: vilanterol.

ICS/LABA/LAMA FDC did not increase the risk of total SAEs (overall RR 1.03, 95% CI 0.98–1.08) vs. dual FDC. However, ICS/LABA/LAMA FDC significantly increased the risk of CV SAEs (RR 1.29, 95% CI 1.10–1.51; *p* < 0.01) vs. ICS/LABA FDC and the risk of pneumonia (RR 1.66, 95% CI 1.42–1.94; *p* < 0.001) vs. LABA/LAMA FDC. ICS/LABA/LAMA FDC also significantly reduced the risk of all-cause mortality (RR 0.75, 95% CI 0.58–0.96; *p* < 0.05) vs. LABA/LAMA FDC (Figure 3A–D).

The sensitivity analysis reported that the KRONOS [30] and ETHOS [14] RCTs introduced substantial heterogeneity on the risk of total and CV SAEs. After resolving heterogeneity, the effect estimates resulting from the sensitivity analysis showed that ICS/LABA/LAMA FDC significantly enhanced the risk of total SAEs (RR 1.05, 95% CI 1.00–1.10; *p* < 0.05) and reduced the risk of CV SAEs (RR 0.66, 95% CI 0.53–0.80; *p* < 0.001) vs. LABA/LAMA FDC (Figure S2A,B).

### 3.3. Network Meta-Analysis

No significant (*p* > 0.05) differences were detected across the different ICS/LABA/LAMA FDCs concerning their impact the risk of moderate to severe AECOPD, trough FEV_1_, TDI, and SGRQ (Figure S3A–D). Furthermore, the impact on the risk of total SAEs, CV SAEs, pneumonia, and all-cause mortality was not significantly (*p* > 0.05) different across the investigated ICS/LABA/LAMA FDCs (Figure S4A–D). Detailed information is shown in Table 2.

**Table 2:** Relative effects with 95% CrI and GRADE score resulting from the overall network meta-analysis; treatment comparisons are sorted in agreement with SUCRA.

Comparisons	Efficacy				Safety			
	Number of Moderate or Severe AECOPD (RR)	Trough FEV_1_ (mL)	TDI (Score)	SGRQ (Score)	Total SAEs (RR)	CV SAEs (RR)	Pneumonia (RR)	All-Cause Mortality (RR)
**BDP/FOR/GLY 200/12/25 µg BID vs.**								
BUD/GLY/FOR 320/18/9.6 µg BID	1.03 (0.25–4.49) ++	−9.28 (−95.15–78.11) ++	−0.09 (−0.65–0.49) ++	−0.53 (−2.89–1.87) ++	0.78 (0.22–2.54) ++	1.02 (0.32–3.33) ++	1.34 (0.50–3.59) +++	1.11 (0.31–3.12) +++
BUD/GLY/FOR 160/18/9.6 µg BID	0.92 (0.19–4.48) ++	0.03 (−93.27–95.63) ++	−0.09 (−0.73–0.47) ++	−0.63 (−3.20–1.89) ++	0.72 (0.19–2.58) +++	0.84 (0.25–2.80) +++	1.54 (0.56–4.39) ++++	0.81 (0.24–2.53) +++
FF/UMEC/VI 100/62.5/25 µg QD	0.86 (0.17–4.41) ++	−29.01 (−121.27–66.14) ++	0.01 (−0.61–0.64) ++	−0.07 (−2.58–2.46) ++	0.68 (0.17–2.51) +++	0.67 (0.20–2.19) +++	1.17 (0.43–3.27) +++	1.07 (0.33–3.20) +++
**BUD/GLY/FOR 320/18/9.6 µg BID vs.**								
BUD/GLY/FOR 160/18/9.6 µg BID	0.89 (0.31–2.63) +++	8.86 (−52.71–71.98) +++	−0.02 (−0.43–0.33) +++	−0.17 (−1.68–1.45) +++	0.93 (0.39–2.30) +++	0.82 (0.37–1.69) +++	1.14 (0.60–2.27) ++++	0.73 (0.37–1.61) ++++
FF/UMEC/VI 100/62.5/25 µg QD	0.83 (0.24–2.92) ++	−20.11 (−90.78–58.07) ++	0.09 (−0.36–0.56) ++	0.47 (−1.27–2.31) ++	0.87 (0.31–2.43) ++	0.65 (0.26–1.49) +++	0.85 (0.43–1.85) +++	0.95 (0.40–2.42) +++
**BUD/GLY/FOR 160/18/9.6 µg BID vs.**								
FF/UMEC/VI 100/62.5/25 µg QD	0.93 (0.22–3.89) ++	−29.36 (−108.70–57.26) ++	0.11 (−0.37–0.66) ++	0.62 (−1.38–2.68) ++	0.94 (0.30–3.05) ++	0.80 (0.30–1.98) ++	0.75 (0.34–1.72) ++++	1.28 (0.55–3.30) ++++

Data are sorted according to the risk of moderate or severe AECOPD as shown in Table 3. Quality of evidence according to GRADE: ++++ high, +++ moderate, ++ low. AECOPD: acute exacerbation of COPD; BDP: beclomethasone dipropionate; BID: bis in die, twice daily; BUD: budesonide; COPD: chronic obstructive pulmonary disease; CV: cardiovascular; FEV_1_: forced expiratory volume in the first second; FF: fluticasone furoate; FOR: formoterol; GLY: glycopyrronium bromide or glycopyrrolate; GRADE: Grading of Recommendations Assessment, Development, and Evaluation; QD: quaque die, once daily; RR: relative risk; SAE: serious adverse event; SGRQ: St. George’s Respiratory Questionnaire; SUCRA: surface under the cumulative ranking curve analysis; TDI: transition dyspnea index; UMEC: umeclidinium bromide; VI: vilanterol; 95% CrI: 95% credible interval.

**Table 3:** SUCRA ^§^ values for efficacy and safety of triple FDCs according to the sensitivity analysis performed on the Bayesian network.

Combinations	Efficacy				Safety			
	Risk of Moderate or Severe AECOPD	Change in Trough FEV_1_	Change in TDI	Change in SGRQ	Risk of Total SAEs	Risk of CV SAEs	Risk of Pneumonia	Risk of All-Cause Mortality
BDP/FOR/GLY 200/12/25 µg BID	0.76	0.67	0.57	0.78	0.87	0.70	0.22	0.55
BUD/GLY/FOR 320/18/9.6 µg BID	0.67	0.76	0.69	0.62	0.38	0.62	0.49	0.70
BUD/GLY/FOR 160/18/9.6 µg BID	0.67	0.56	0.74	0.56	0.33	0.44	0.67	0.34
FF/UMEC/VI 100/62.5/25 µg QD	0.66	0.73	0.67	0.79	0.22	0.39	0.30	0.65

^§^ SUCRA = 1 when a treatment is considered to be the best, and SUCRA = 0 when a treatment is considered to be the worst; the SUCRA values were divided by quartiles where a score of 0–0.25 is the lowest quartile and 0.75–1.00 is the highest quartile. AECOPD: acute exacerbation of COPD; BDP: beclomethasone dipropionate; BID: bis in die, twice daily; BUD: budesonide; COPD: chronic obstructive pulmonary disease; CV: cardiovascular; FEV_1_: forced expiratory volume in the first second; FDC: fixed-dose combination; FF: fluticasone furoate; FOR: formoterol; GLY: glycopyrronium bromide or glycopyrrolate; QD: quaque die, once daily; SAE: serious adverse event; SGRQ: St. George’s Respiratory Questionnaire; SUCRA: surface under the cumulative ranking curve; TDI: transition dyspnea index; UMEC: umeclidinium bromide; VI: vilanterol.

**Figure 4:**
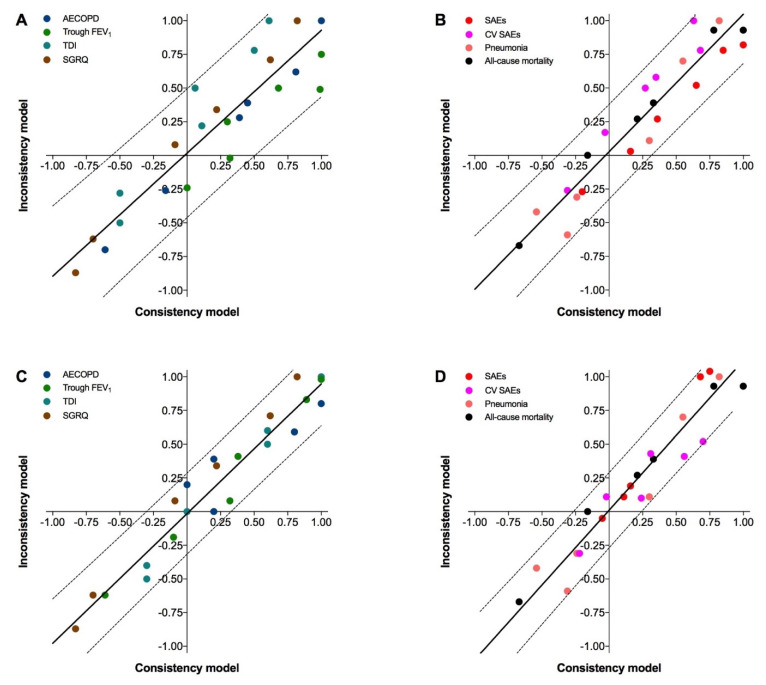
Assessment of the risk of bias via the consistency/inconsistency regression with 95% prediction bands concerning the efficacy (**A**) and safety (**B**) outcomes and after sensitivity analysis (**C**,**D**) by excluding the treatment comparisons introducing inconsistency in the Bayesian network. AECOPD: acute exacerbation of COPD; CV: cardiovascular; FEV_1_: forced expiratory volume in the first second; SAE: serious adverse event; SGRQ: St. George’s Respiratory Questionnaire; TDI: transitional dyspnea index.

The consistency/inconsistency analysis reported the presence of bias in the Bayesian network of efficacy (*R*^2^ 0.844, Sy.x 0.225) and safety (*R*^2^ 0.905, Sy.x 0.165) (Figure 4A,B). The sensitivity analysis confirmed that the same treatment comparisons in the KRONOS [30] and IMPACT [34] RCTs that caused substantial heterogeneity in the pairwise meta-analysis also introduced significant (*p* < 0.05) inconsistency in the network meta-analysis. Removing these treatment comparisons reduced the risk of bias in the Bayesian network (efficacy: *R*^2^ 0.938, Sy.x 0.142; safety: *R*^2^ 0.917, Sy.x 0.158) (Figure 4C,D). The analysis of residual plot confirmed this trend (Figure S5A,B).

### 3.4. SUCRA

In order to improve the accuracy of the network meta-analysis, the SUCRA and IBiS score were calculated on data resulting from the sensitivity analysis.

The SUCRA reported a similar trend in the efficacy profile across the investigated triple FDCs with respect to their impact on the risk of moderate or severe AECOPD, as well as the improvement in trough FEV_1_, TDI, and SGRQ; overall, the SUCRA values resulted always in the upper two quartiles (first and second). Concerning the safety profile, the SUCRA value of total SAEs for BDP/FOR/GLY 200/12/25 µg BID was in the upper quartile (first), whereas that of the other triple FDCs resulted in the lower quartiles (third and fourth). The SUCRA values regarding the risk of CV SAEs resulted in the second quartile for BDP/FOR/GLY 200/12/25 µg BID and BUD/GLY/FOR 320/18/9.6, whereas the SUCRA value for BUD/GLY/FOR 160/18/9.6 µg BID and FF/UMEC/VI 100/62.5/25 µg QD was in the third. The SUCRA value concerning the risk of pneumonia was greater for BUD/GLY/FOR 160/18/9.6 µg BID (second) than for the other triple FDCs (third and fouth). Considering the risk of all-cause mortality, BUD/GLY/FOR 160/18/9.6 µg BID resulted in a lower SUCRA value (third quartile) than the other triple FDCs (second quartile). Detailed information on the SUCRA values and differences in quartiles for the efficacy and safety profile of triple FDCs are shown in Table 3.

### 3.5. IBiS

**Figure 5:**
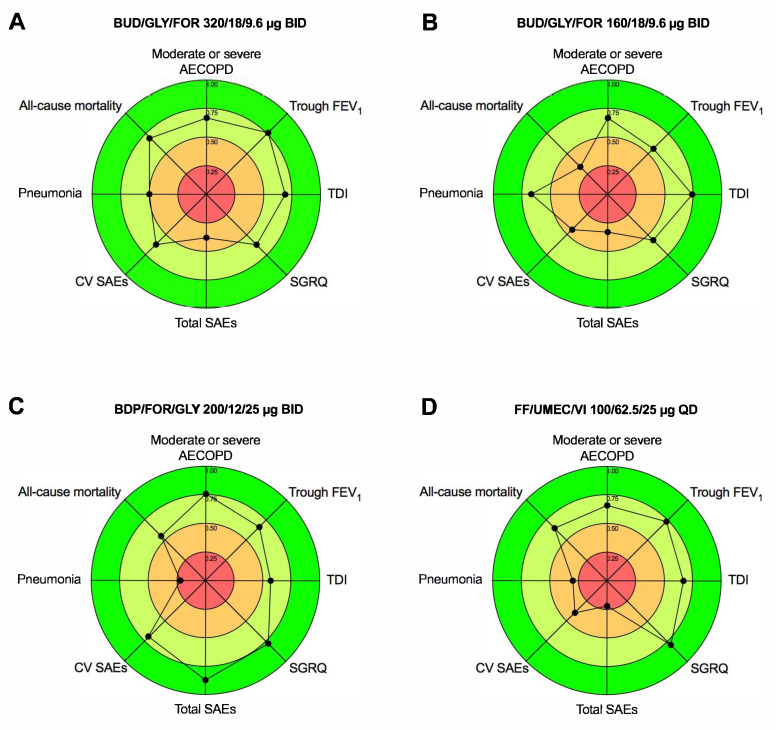
Graphical representation of combined efficacy/safety profile of BUD/GLY/FOR 320/18/9.6 µg BID (**A**), BUD/GLY/FOR 160/18/9.6 µg BID (**B**), BDP/FOR/GLY 200/12/25 µg BID (**C**), and FF/UMEC/VI 100/62.5/25 µg QD (**D**) in COPD patients according to the IBiS score; a greater area indicates a better efficacy/safety profile. AECOPD: acute exacerbation of COPD; BDP: beclomethasone dipropionate; BID: bis in die, twice daily; BUD: budesonide; COPD chronic obstructive pulmonary disease; CV: cardiovascular; FDC: fixed-dose combination; FEV_1_: forced expiratory volume in the first second; FF: fluticasone furoate; FOR: formoterol fumarate; GLY: glycopyrronium bromide or glycopyrrolate; ICS: inhaled corticosteroid; IBiS: Implemented Bidimensional SUCRA; LABA: long-acting β_2_-adrenoceptor agonist; LAMA: long-acting muscarinic antagonist; QD: quaque die, once daily; SAEs: serious adverse events; SGRQ: St. George’s Respiratory Questionnaire; SUCRA: surface under the cumulative ranking curve; TDI: transitional dyspnea index; UMEC: umeclidinium bromide; VI: vilanterol.

The combined efficacy/safety profile resulting from the IBiS score (Figure 5) provided the following rank: BDP/FOR/GLY 200/12/25 µg BID (area 41.41%) ⪞ BUD/GLY/FOR 320/18/9.6 µg BID (area 38.27%) ⪞ FF/UMEC/VI 100/62.5/25 µg QD (area 36.26%) > BUD/GLY/FOR 160/18/9.6 µg BID (area 32.62%). The specific IBiS analysis on efficacy and safety profile showed that FF/UMEC/VI 100/62.5/25 µg QD was the most effective FDC, and BDP/FOR/GLY 200/12/25 µg BID was the safest FDC (detailed information reported in Figures S6 and S7).

### 3.6. Bias and Quality of Evidence

The quality of evidence was consistently low for all the efficacy outcomes. With respect to the safety profile, the quality of evidence was low to moderate for total SAEs, CV SAEs, and all-cause mortality, whereas a moderate to high quality of evidence was detected for pneumonia. The weighted and traffic light plots are reported in Figure S8, and the GRADE scores are shown in Table 2. Further detailed information can be found in the Supplementary Materials.

## 4. Discussion

The results of this meta-analysis suggest that the investigated ICS/LABA/LAMA FDCs are equally effective in the treatment of COPD, with no significant difference concerning their impact on AECOPD, lung function, dyspnea, and quality of life, regardless of the ICS dose and regimen of administration (i.e., twice daily vs. once daily). The IBiS score generally confirmed the benefits of ICS/LABA/LAMA FDCs on efficacy outcomes, detecting that the effect against the risk of AECOPD was not related to the level of ICS in the FDC. Conversely, it resulted that BDP/FOR/GLY 160/18/9.6 µg BID, the only FDC including an ICS at lower dose, was ranked as the less effective in improving trough FEV_1_.

Interestingly, all the ICS/LABA/LAMA FDCs were also characterized by similar favorable safety profiles, with no significant difference in the risk of total SAEs, CV SAEs, pneumonia, and all-cause mortality. However, the IBiS score indicated that the formulation with a lower dose of ICS (i.e., BDP/FOR/GLY 160/18/9.6 µg BID) had an advantage with respect to the risk of pneumonia over the other FDCs including higher ICS dose, further supporting the large body evidence that, in COPD patients, the risk of pneumonia is directly related to the dose of ICS [35]. To the best of our knowledge, this finding provides for the first time the evidence that, although there may be a certain level of risk of pneumonia in patients treated with ICS/LABA/LAMA FDCs, such a risk seems to be not associated with a specific ICS, but is directly related to the ICS dose included in the FDC. On the other hand, the IBiS score evidenced that all the ICS/LABA/LAMA FDCs including an ICS at higher dose resulted in greater rank with respect to the protection against the risk of all-cause mortality than that including a lower dose of ICS. This evidence confirms the hypothesis that the reduced risk of all-cause mortality may be due to a CV protective effect of the ICS, as previously suggested [18]. In fact, focusing on the safety profile of the only FDC tested at two different doses of ICS, it is evident that the reduced risk of all-cause mortality detected for BUD/GLY/FOR 320/18/9.6 was accompanied by a reduced risk of CV SAEs, whereas the higher risk of all-cause mortality detected for BUD/GLY/FOR 160/18/9.6 was accompanied by a higher risk of CV SAEs.

Although from a strict statistical point of view the comparison across the triple FDCs may seem a moot point due to the lack of significant differences, we have to highlight that the IBiS score provides clinically important information that allows optimizing the therapeutic approach of severe COPD patients requiring an ICS/LABA/LAMA FDC. Withdrawal from ICS has been extensively demonstrated to have a negative impact on lung function in patients previously treated with triple combination therapy [36,37]; here, we provide the evidence that a reduced dose of ICS in the triple FDC may also not be optimal in terms of trough FEV_1_. Outside of this peculiarity, the network meta-analysis suggests that the real challenge among BDP/FOR/GLY, BUD/GLY/FOR, and FF/UMEC/VI is in the safety profile. In this regard, in subjects with a history of pneumonia, it would be better to administer a triple FDC including the ICS at lower dose; conversely, in those patients at high CV risk, any ICS/LABA/LAMA FDCs including an ICS at higher dose may be chosen to protect from all-cause mortality.

A large body of evidence indicates that the monocomponents included in the triple formulations are characterized by specific PK and PD properties [5,6,7,8]. Nevertheless, it seems that the subtle, but potentially clinically relevant difference between the triple FDCs may be prevalently related to the dose level of the ICS, at least with respect to the efficacy profile. Thus, the failure of clinical benefits with a specific ICS/LABA/LAMA FDC should probably preclude a trial of other triple FDCs. Indeed combining an ICS with a LABA and a LAMA elicits beneficial synergistic interaction, and modulating the dose of the ICS when the LABA and the LAMA are correctly balanced [38] may lead to improvement in airflow obstruction and bronchial inflammation [39,40]. Unfortunately, to date, no triple FDCs including an ICS at lower dose have been approved in COPD, providing a missing opportunity for a tailored therapy in those patients prone to pneumonia.

The current GOLD recommendations [1] suggest using an ICS/LABA/LAMA FDC only at follow-up in COPD patients with persistent breathlessness/exercise limitation or persistent exacerbations already taking maintenance treatments with an ICS/LABA FDC or a LABA/LAMA FDC. In this regard, the results originating from the pairwise meta-analysis are mainly supportive of previous findings [41,42], providing evidence that adding an ICS to LABA/LAMA is more effective in preventing AECOPD than adding a LAMA to ICS/LABA; conversely, as expected, adding a LAMA to ICS/LABA was more effective in improving trough FEV_1_ than adding an ICS to LABA/LAMA. Overall, these findings support the GOLD [1] strategy at follow-up, highlighting the pivotal role of the ICS especially in patients with a history of AECOPD and high blood eosinophil count. The pairwise comparing ICS/LABA/LAMA FDC with dual bronchodilator therapy further supports the protective effect of the ICS in the combination with respect to the risk of CV SAEs and mortality, although at cost of a higher risk of pneumonia.

In this study, we resolved the intrinsic limitations typical of the meta-analysis techniques [43] by providing ranks resulting from unbiased effect estimates in which heterogeneity and inconsistency were resolved. Nevertheless, the main limitation of this meta-analysis was anchored in the methodological matter typical of the network approach when active treatments are indirectly compared to each other, a condition that unfortunately cannot be solved by the currently available treatment comparisons [44]. Effectively, no studies have directly compared different ICS/LABA/LAMA FDCs in the same RCT. Thus, although the comparisons across the RCTs included in this meta-analysis were based on consistency assumption according with Bayesian statistics [45], the comparisons across BDP/FOR/GLY, BUD/GLY/FOR, and FF/UMEC/VI unfortunately remain indirect. As a matter of fact, excluding the links between the KRONOS [30] and ETHOS [31] RCTs, the dual FDCs used as common links among the nodes were the same as the triple FDCs within but not across the studies, a limitation not related to our analysis but to the lack of comparative studies. Certainly, with an increasing number of links distancing the interventions to be compared, indirect comparisons become less reliable, thus affecting the degree of power and precision of indirect evidence [44,45]. The availability of direct comparisons from RCTs would have improved the robustness of the network and the quality of evidence, especially when considering the efficacy profile. Similarly, whether a BID is better than a QD formulation, or vice versa, would be better assessed by a comparative effectiveness study rather than an efficacy study. However, this was not the case; since there is probably no interest in performing RCTs that directly compare ICS/LABA/LAMA FDCs, well-performed network meta-analysis remains the only tool for evidence-based medicine with the current higher level of evidence from the current available research [45,46].

Lastly, since only four studies passed the strict inclusion criteria of our protocol, it was not possible to carry out subset analyses in specific COPD populations to assess if ICS/LABA/LAMA FDCs may really be considered a precision medicine tool.

Concluding, the comparison of the efficacy and safety profile across the currently available ICS/LABA/LAMA FDCs in COPD should be interpreted with caution and according to the degree of certainty resulting from the level of evidence. Indeed, this meta-analysis can represent a lens through which evidence on the maximization of inhaled therapy in COPD may be viewed [47], highlighting the strong need for head-to-head RCTs comparing BDP/FOR/GLY, BUD/GLY/FOR, and FF/UMEC/VI to give clinicians the opportunity to identify the best triple FDC to treat the specific clinical traits of each single COPD patient and reduce the risk of potential SAEs.

## Figures and Tables

**Table 1:** Main characteristics of the RCTs included in the network meta-analysis.

Author, Year, Clinical Trial Identifier, Study Name, and Reference	Trial Characteristics	Duration of Treatment (Weeks)	Number of Analyzed Patients	Drugs, Doses, Regimen of Administration, Device	Main Inclusion Criteria	Age (Years)	Male (%)	Current Smokers (%)	Smoking History (Pack-Years)	Post Bronchodilator FEV_1_ (% Predicted)	Reversibility (% Patients)	Patient with AECOPD History (%)	AECOPD in the Previous Year (Rate)	Blood Eosinophil Count at Baseline (Cells per µL)	Blood Eosinophils Subgroups (Cells per µL)	Jadad Score
Rabe et al., 2021, NCT02465567, ETHOS pulmonary function test sub-study [31]	Phase III, randomized, double-blind, parallel-group, active control, multicenter	52	3088	BUD/GLY/FOR (320/18/9.6 μg BID via MDI); BUD/GLY/FOR (160/18/9.6 μg BID via MDI); GLY/FOR (18/9.6 μg BID via MDI); BUD/FOR (320/9.6 μg BID via MDI)	Pre-bronchodilator FEV_1_ < 65% predicted	64.4	47.2	44.0	43.9	42.8	34.1	100.0	1.5	NA	<150; ≥150	4
Rabe et al., 2020, NCT02465567, ETHOS [14,33]	Phase III, randomized, double-blind, parallel-group, active control, multicenter	52	8509	BUD/GLY/FOR (320/18/9.6 μg BID via MDI); BUD/GLY/FOR (160/18/9.6 μg BID via MDI); GLY/FOR (18/9.6 μg BID via MDI); BUD/FOR (320/9.6 μg BID via MDI)	Post-bronchodilator FEV_1_ ≥ 25% and ≤65% predicted	64.7	59.7	41.1	47.6	43.4	30.7	100.0	1.7	167	<150; ≥150	4
Tabberer et al., 2020, NCT02164513, IMPACT sub-study [32]	Phase III, randomized, double-blind, parallel-group, active control, multicenter	52	5058	FF/UMEC/VI (100/62.5/25 μg QD via DPI); UMEC/VI (62.5/25 μg QD via DPI); FF/VI (100/25 μg QD via DPI)	(a) Post-bronchodilator FEV_1_ < 50% predicted and ≥1 moderate or severe AECOPD in the previous year; (b) post-bronchodilator FEV_1_ ≥ 50% and ≤80% predicted and ≥2 moderate or ≥1 severe AECOPD in the previous year	64.7	56.0	NA	NA	NA	NA	NA	NA	NA	NA	3
Ferguson et al., 2018, NCT02497001, KRONOS [30]	Phase III, randomized, double-blind, parallel-group, active control, multicenter	24	1578	BUD/GLY/FOR (320/18/9.6 μg BID via MDI); GLY/FOR (18/9.6 μg BID via MDI); BUD/FOR (320/9.6 μg BID via MDI)	Post-bronchodilator FEV_1_ ≥ 25% and ≤80% predicted	65.1	70.6	39.3	45.0	50.1	42.9	25.4	0.3	153	<150; ≥150	5
Lipson et al., 2018, NCT02164513, IMPACT [15,34]	Phase III, randomized, double-blind, parallel-group, active control, multicenter	52	10,355	FF/UMEC/VI (100/62.5/25 μg QD via DPI); UMEC/VI (62.5/25 μg QD via DPI); FF/VI (100/25 μg QD via DPI)	(a) Post-bronchodilator FEV_1_ < 50% predicted and ≥1 moderate or severe AECOPD in the previous year; (b) post-bronchodilator FEV_1_ ≥ 50% and ≤80% predicted and ≥2 moderate or ≥1 severe AECOPD in the previous year	65.3	66.0	35.0	≥10.0	45.5	18.0	100.0	1.7	≃150	<150; ≥150	3
Singh et al., 2016, NCT01917331, TRILOGY [16]	Phase III, randomized, double-blind, parallel-group, active control, multicenter	52	1367	BDP/FOR/GLY (200/12/25 μg BID via MDI); BDP/FOR (200/12 μg BID via MDI)	Post-bronchodilator FEV_1_ < 50% predicted and ≥1 moderate or severe AECOPD in the previous year	63.6	75.5	47.0	≥10.0	36.6	NA	100.0	1.2	245	<200; ≥200	5

Reversibility was defined as an increase in FEV_1_ of ≥12% and >200 mL following administration of salbutamol. AECOPD: acute exacerbation of COPD; BID: bis in die, twice daily; BDP: beclomethasone dipropionate; BUD: budesonide; COPD: chronic obstructive pulmonary disease; DPI: dry-powder inhaler; FEV_1_: forced expiratory volume in the first second; FOR: formoterol fumarate; FF: fluticasone furoate; GLY: glycopyrronium bromide or glycopyrrolate; MDI: metered-dose inhaler; NA: not available; QD: quaque die, once daily; RCT: randomized controlled trial; UMinconsistency in network metaRisk-of-bias VISuaEC: umeclidinium bromide; VI: vilanterol.

## Data Availability

Not applicable.

## Supplementary Materials

The following supporting information can be downloaded at: https://www.mdpi.com/article/10.3390/jcm11154491/s1, Table S1. PRISMA-P Checklist. Table S2. Definition of moderate or severe AECOPD as reported in the RCTs included in the network meta-analysis. Figure S1. Forest plots of the sensitivity analysis performed by excluding the comparisons that introduced substantial heterogeneity in the overall pairwise meta-analysis of efficacy profile. Figure S2. Forest plots of the sensitivity analysis performed by excluding the comparisons that introduced substantial heterogeneity in the overall pairwise meta-analysis of safety profile. Figure S3. Forest plots of the overall network meta-analysis of efficacy profile. Figure S4. Forest plots of the overall network meta-analysis of safety profile. Figure S5. Residual plot of the overall consistency/inconsistency regression before (A) and after sensitivity analysis (B) to reduce the risk of bias in the overall Bayesian network. Figure S6. Graphical representation of efficacy profile of ICS/LABA/LAMA FDCs in COPD patients according the IBiS score: the greater the area, the better the efficacy profile. Figure S7. Graphical representation of safety profile of ICS/LABA/LAMA FDCs in COPD patients according the IBiS score: the greater the area, the better the safety profile. Figure S8. Assessment of the risk of bias via the weighted plot for the assessment of the overall risk of bias (A) and the traffic light plot of the risk of bias of each included RCT via the Cochrane RoB 2 tool (B) (*n* = 4 studies). Traffic light plot reports five risk of bias domains: D1, bias arising from the randomization process; D2, bias due to deviations from intended intervention; D3, bias due to missing outcome data; D4, bias in measurement of the outcome; D5, bias in selection of the reported result; Yellow circle indicates some concerns on the risk of bias and green circle represents low risk of bias. References [48,49,50,51,52,53,54,55,56] are cited in the Supplementary Materials.
