# Supplementary material for: Comparing the Efficacy and Safety Profile of Triple Fixed-Dose Combinations in COPD: A Meta-Analysis and IBiS Score
Source: J Clin Med. 2022 Aug 1;11(15):4491. doi: 10.3390/jcm11154491 (PMC9369741; doi:10.3390/jcm11154491)
Supplement: Supplementary file 1 [file jcm-11-04491-s001.zip › jcm-1808073-supplementary.pdf]

Supplement

Methods

Search strategy

Research string: COPD, triple, Most Recent, Randomized Controlled Trial,"(("pulmonary disease, chronic obstructive"[MeSH Terms] OR ("pulmonary"[All Fields] AND "disease"[All Fields] AND "chronic"[All Fields] AND "obstructive"[All Fields]) OR "chronic obstructive pulmonary disease"[All Fields] OR "copd"[All Fields]) AND ("triple"[All Fields] OR "triples"[All Fields])) AND ((randomizedcontrolledtrial[Filter])".

Literature search results were uploaded to Eppi-Reviewer 4 (EPPI-Centre Software, London, UK), a web-based software program for managing and analysing data in literature reviews that facilitates collaboration among reviewers during the study selection process.

Data extraction

The inter- and intra-rater reliability for data abstraction was assessed via the Cohen's Kappa score, as previously described [1]. Briefly, Cohen's Kappa ≥0.80 indicated excellent agreement, coefficients between 0.61 and 0.80 represented substantial agreement, coefficients between 0.41 and 0.61 moderate agreement and <0.41 fair to poor agreement.

Data synthesis and analysis

A full Bayesian evidence network was used in the network meta-analysis (chains: 4; initial values scaling: 2.5; tuning iterations: 20.000; simulation iterations: 50.000; tuning interval: 10). The convergence diagnostics for consistency and inconsistency were assessed via the Brooks-Gelman-Rubin method, as previously described [2]. Due to the characteristics of parameters besides the available data, the just proper non-informative distributions specified the prior densities, in agreement with the Bayesian Approaches to Clinical Trials and Health-Care Evaluation [3,4]. Since the distributions were sufficiently vague, the reference treatment, study baseline effects, and heterogeneity variance were unlikely to have a noticeable impact on model results. In this condition, GeMTC software automatically generates and runs the required Bayesian hierarchical model and selects the prior distributions and starting values as well, via heuristically determining a value for the outcome scale parameter (i.e. outcome scale S) [5,6]. The posterior mean deviance of data points in the unrelated mean effects model was plotted against their posterior mean deviance in the consistency model in order to provide information for identifying the loops in the treatment network where evidence was inconsistent [7].

Quality of studies, risk bias, and evidence profile

The Jadad score ranges from 1 to 5 (score of 5 being the best score), and the quality of studies was ranked as follows: score <3, low quality; score =3, medium quality; score >3 high quality. The weighted assessment of the risk of bias was analyzed via the Cochrane RoB 2 [8].

The normalized consistency/inconsistency analysis is a procedure that allows assessing whether the outcomes resulting from the consistency and inconsistency models fit adequately with the line of equality, as previously described [9]. The residual plot analysis was carried out on the consistency/inconsistency regression to check for the goodness of fit.

Quality of evidence according to GRADE: ++++ high quality, +++ moderate quality, ++ low quality, + very low quality [8].

Software and statistical significance

ImageJ was used to extract data from the figures, when necessary [10], OpenMeta-Analyst [11] and GeMTC [12] were used to perform the meta-analyses, GraphPad Prism (CA, US) to graph the data, GRADEpro GDT to assess the quality of evidence [8], and the robvis visualization software to perform the RoB 2 tool [13,14].

Results

Study characteristics

Data from 21,809 COPD patients (44.64% treated with ICS/LABA/LAMA FDC, 33.28% treated with ICS/LABA FDC, 22.08% treated with LABA/LAMA FDC) were extracted from the ETHOS [15], IMPACT [16], KRONOS [17], and TRILOGY [18] Phase III RCTs published as full-text papers between 2016 and 2020. The sub-studies of ETHOS [19] and IMPACT [20] were included as well, since they provided additional data concerning lung function and quality of life respectively, which were missing in the primary articles. For the KRONOS [17] and TRILOGY [18] RCTs, data on all-cause mortality were extracted from primary publications, whereas those from the ETHOS [21] and IMPACT [22] were extracted from the final retrieved datasets. The inter-rater reliability for data abstraction was excellent before and after the learning process (Cohen's Kappa 0.96 and 1.00, respectively). The intra-rater reliability produced a Cohen's Kappa of 1.00 after the learning process.

Bias and quality of evidence

Two RCTs out of four had some concerns on the risk of bias in the domains of randomization process (50.0%) and measurement of the outcome (50.0%). One RCT had some concerns

for deviations from intended intervention (25.0%) while the remaining three studies presented a low risk of bias for the domain (75.0%). All four RCTs had a low risk of bias due to missing outcome (100.0%) and selection of the reported results (100.0%).

References

[1] M.E. Gianinazzi, C.S. Rueegg, K. Zimmerman, C.E. Kuehni, G. Michel, G. Swiss Paediatric Oncology, Intra-rater and inter-rater reliability of a medical record abstraction study on transition of care after childhood cancer, PLoS One. 10 (2015) e0124290. https://doi.org/10.1371/journal.pone.0124290.

[2] L. Calzetta, P. Rogliani, J. Ora, E. Puxeddu, M. Cazzola, M.G. Matera, LABA/LAMA combination in COPD: a meta-analysis on the duration of treatment, Eur. Respir. Rev. 26 (2017) 160043.

[3] G. Lu, A.E. Ades, Assessing evidence inconsistency in mixed treatment comparisons, J. Am. Stat. Assoc. 101 (2006) 447–459.

[4] D.J. Spiegelhalter, K.R. Abrams, J.P. Myles, Bayesian approaches to clinical trials and health-care evaluation, John Wiley & Sons, 2004.

[5] G. van Valkenhoef, G. Lu, B. de Brock, H. Hillege, A.E. Ades, N.J. Welton, Automating network meta-analysis, Res Synth Methods. 3 (2012) 285–299. https://doi.org/10.1002/jrsm.1054.

[6] G. van Valkenhoef, S. Dias, A.E. Ades, N.J. Welton, Automated generation of node-splitting models for assessment of inconsistency in network meta-analysis, Res. Synth. Methods. 7 (2016) 80–93.

[7] S. Dias, N.J. Welton, A.J. Sutton, D.M. Caldwell, G. Lu, A.E. Ades, Evidence synthesis for decision making 4: inconsistency in networks of evidence based on randomized controlled trials, Med. Decis. Mak. 33 (2013) 641–656.

[8] G. Guyatt, A.D. Oxman, E.A. Akl, R. Kunz, G. Vist, J. Brozek, S. Norris, Y. Falck-Ytter, P. Glasziou, H. DeBeer, R. Jaeschke, D. Rind, J. Meerpohl, P. Dahm, H.J. Schunemann, GRADE guidelines: 1. Introduction-GRADE evidence profiles and summary of findings tables, J Clin Epidemiol. 64 (2011) 383–394. https://doi.org/10.1016/j.jclinepi.2010.04.026.

[9] M. Cazzola, P. Rogliani, L. Calzetta, N.A. Hanania, M.G. Matera, Impact of Mucolytic Agents on COPD Exacerbations: A Pair-wise and Network Meta-analysis, COPD J. Chronic Obstr. Pulm. Dis. 14 (2017). https://doi.org/10.1080/15412555.2017.1347918.

[10] M.D. Abràmoff, P.J. Magalhães, S.J. Ram, Image processing with ImageJ, Biophotonics Int. 11 (2004) 36–42.

[11] B.C. Wallace, I.J. Dahabreh, T.A. Trikalinos, J. Lau, P. Trow, C.H. Schmid, Closing the gap between methodologists and end-users: R as a computational back-end, J Stat Softw. 49 (2012) 1–15.

[12] G. van Valkenhoef, G. Lu, B. de Brock, H. Hillege, A.E. Ades, N.J. Welton, Automating network meta-analysis, Res. Synth. Methods. 3 (2012) 285–299. https://doi.org/10.1002/jrsm.1054.

[13] J.A.C. Sterne, J. Savovic, M.J. Page, R.G. Elbers, N.S. Blencowe, I. Boutron, C.J. Cates, H.Y.

Cheng, M.S. Corbett, S.M. Eldridge, J.R. Emberson, M.A. Hernan, S. Hopewell, A. Hrobjartsson, D.R. Junqueira, P. Juni, J.J. Kirkham, T. Lasserson, T. Li, A. McAleenan, B.C. Reeves, S. Shepperd, I. Shrier, L.A. Stewart, K. Tilling, I.R. White, P.F. Whiting, J.P.T. Higgins, RoB 2: a revised tool for assessing risk of bias in randomised trials, Bmj. 366 (2019) l4898. https://doi.org/10.1136/bmj.l4898.

[14] L.A. McGuinness, J.P.T. Higgins, Risk-of-bias VISualization (robvis): An R package and shiny web app for visualizing risk-of-bias assessments, Res. Syn. Meth. (2020) 1–7.

[15] K.F. Rabe, F.J. Martinez, G.T. Ferguson, C. Wang, D. Singh, J.A. Wedzicha, R. Trivedi, E. St. Rose, S. Ballal, J. McLaren, P. Darken, M. Aurivillius, C. Reisner, P. Dorinsky, Triple Inhaled Therapy at Two Glucocorticoid Doses in Moderate-to-Very-Severe COPD, N. Engl. J. Med. 383 (2020) 35–48. https://doi.org/10.1056/nejmoa1916046.

[16] D.A. Lipson, F. Barnhart, N. Brealey, J. Brooks, G.J. Criner, N.C. Day, M.T. Dransfield, D.M.G. Halpin, M.K. Han, C.E. Jones, S. Kilbride, P. Lange, D.A. Lomas, F.J. Martinez, D. Singh, M. Tabberer, R.A. Wise, S.J. Pascoe, Once-Daily Single-Inhaler Triple versus Dual Therapy in Patients with COPD, N. Engl. J. Med. 378 (2018) 1671–1680. https://doi.org/10.1056/NEJMoa1713901.

[17] G.T. Ferguson, K.F. Rabe, F.J. Martinez, L.M. Fabbri, C. Wang, M. Ichinose, E. Bourne, S. Ballal, P. Darken, K. DeAngelis, M. Aurivillius, P. Dorinsky, C. Reisner, Triple therapy with budesonide/glycopyrrolate/formoterol fumarate with co-suspension delivery technology versus dual therapies in chronic obstructive pulmonary disease (KRONOS): a double-blind, parallel-group, multicentre, phase 3 randomised controlled trial, Lancet Respir. Med. 6 (2018) 747–758. https://doi.org/10.1016/S2213-2600(18)30327-8.

[18] D. Singh, A. Papi, M. Corradi, I. Pavlišová, I. Montagna, C. Francisco, G. Cohuet, S. Vezzoli, M. Scuri, J. Vestbo, Single inhaler triple therapy versus inhaled corticosteroid plus long-acting β2-agonist therapy for chronic obstructive pulmonary disease (TRILOGY): a double-blind, parallel group, randomised controlled trial, Lancet. 388 (2016) 963–973. https://doi.org/10.1016/S0140-6736(16)31354-X.

[19] K.F. Rabe, F.J. Martinez, D. Singh, R. Trivedi, M. Jenkins, P. Darken, M. Aurivillius, P. Dorinsky, Improvements in lung function with budesonide/glycopyrrolate/formoterol fumarate metered dose inhaler versus dual therapies in patients with COPD: a sub-study of the ETHOS trial, Ther. Adv. Respir. Dis. 15 (2021) 175346662110343. https://doi.org/10.1177/17534666211034329.

[20] M. Tabberer, C.E. Jones, S. Kilbride, D.M.G. Halpin, D.A. Lomas, S. Pascoe, D. Singh, R.A. Wise, G.J. Criner, P. Lange, M.T. Dransfield, M.L.K. Han, F.J. Martinez, M.C. Kaisermann, D.A. Lipson, Single-Inhaler Triple Therapy and Health-Related Quality of Life in COPD: The IMPACT Study, Adv. Ther. 37 (2020) 3775–3790. https://doi.org/10.1007/s12325-020-01409-8.

[21] F.J. Martinez, K.F. Rabe, G.T. Ferguson, J.A. Wedzicha, D. Singh, C. Wang, K. Rossman, E. St. Rose, R. Trivedi, S. Ballal, P. Darken, M. Aurivillius, C. Reisner, P. Dorinsky, Reduced All-Cause Mortality in the ETHOS Trial of Budesonide/Glycopyrrolate/Formoterol for COPD: A Randomized, Double-Blind, Multi-Center Parallel-Group Study, Am. J. Respir. Crit. Care Med.

203 (2020). https://doi.org/10.1164/rccm.202006-2618oc.

[22] D.A. Lipson, C. Crim, G.J. Criner, N.C. Day, M.T. Dransfield, D.M.G. Halpin, M.L.K. Han, C.E. Jones, S. Kilbride, P. Lange, D.A. Lomas, S. Lettis, P. Manchester, N. Martin, D. Midwinter, A. Morris, S.J. Pascoe, D. Singh, R.A. Wise, F.J. Martinez, Reduction in All-Cause Mortality with Fluticasone Furoate/Umeclidinium/Vilanterol in Patients with Chronic Obstructive Pulmonary Disease, Am. J. Respir. Crit. Care Med. 201 (2020) 1508–1516. https://doi.org/10.1164/RCCM.201911-2207OC.

Supplementary Tables

Table S1. PRISMA-P Checklist

Section and Topic	Item #	Checklist item	Location where item is reported
**TITLE**			
Title	1	Identify the report as a systematic review.	1
**ABSTRACT**			
Abstract	2	See the PRISMA 2020 for Abstracts checklist.	3
**INTRODUCTION**			
Rationale	3	Describe the rationale for the review in the context of existing knowledge.	4
Objectives	4	Provide an explicit statement of the objective(s) or question(s) the review addresses.	4, 5
**METHODS**			
Eligibility criteria	5	Specify the inclusion and exclusion criteria for the review and how studies were grouped for the syntheses.	5, 6
Information sources	6	Specify all databases, registers, websites, organisations, reference lists and other sources searched or consulted to identify studies. Specify the date when each source was last searched or consulted.	5
Search strategy	7	Present the full search strategies for all databases, registers and websites, including any filters and limits used.	5
Selection process	8	Specify the methods used to decide whether a study met the inclusion criteria of the review, including how many reviewers screened each record and each report retrieved, whether they worked independently, and if applicable, details of automation tools used in the process.	5, 6
Data collection process	9	Specify the methods used to collect data from reports, including how many reviewers collected data from each report, whether they worked independently, any processes for obtaining or confirming data from study investigators, and if applicable, details of automation tools used in the process.	6
Data items	10a	List and define all outcomes for which data were sought. Specify whether all results that were compatible with each outcome domain in each study were sought (e.g. for all measures, time points, analyses), and if not, the methods used to decide which results to collect.	6
	10b	List and define all other variables for which data were sought (e.g. participant and intervention characteristics, funding sources). Describe any assumptions made about any missing or unclear information.	6
Study risk of bias assessment	11	Specify the methods used to assess risk of bias in the included studies, including details of the tool(s) used, how many reviewers assessed each study and whether they worked independently, and if applicable, details of automation tools used in the process.	7
Effect measures	12	Specify for each outcome the effect measure(s) (e.g. risk ratio, mean difference) used in the synthesis or presentation of results.	6, 7
Synthesis methods	13a	Describe the processes used to decide which studies were eligible for each synthesis (e.g. tabulating the study intervention characteristics and comparing against the planned groups for each synthesis (item #5)).	6, 7
	13b	Describe any methods required to prepare the data for presentation or synthesis, such as handling of missing summary statistics, or data conversions.	6, 7
	13c	Describe any methods used to tabulate or visually display results of individual studies and syntheses.	6, 7
	13d	Describe any methods used to synthesize results and provide a rationale for the choice(s). If meta-analysis was performed, describe the model(s), method(s) to identify the presence and extent of statistical heterogeneity, and software package(s) used.	6, 7

Section and Topic	Item #	Checklist item	Location where item is reported
	13e	Describe any methods used to explore possible causes of heterogeneity among study results (e.g. subgroup analysis, meta-regression).	6, 7
	13f	Describe any sensitivity analyses conducted to assess robustness of the synthesized results.	6, 7
Reporting bias assessment	14	Describe any methods used to assess risk of bias due to missing results in a synthesis (arising from reporting biases).	6, 7
Certainty assessment	15	Describe any methods used to assess certainty (or confidence) in the body of evidence for an outcome.	6, 7
**RESULTS**			
Study selection	16a	Describe the results of the search and selection process, from the number of records identified in the search to the number of studies included in the review, ideally using a flow diagram.	7, 8
	16b	Cite studies that might appear to meet the inclusion criteria, but which were excluded, and explain why they were excluded.	7, 8
Study characteristics	17	Cite each included study and present its characteristics.	7, 8
Risk of bias in studies	18	Present assessments of risk of bias for each included study.	10
Results of individual studies	19	For all outcomes, present, for each study: (a) summary statistics for each group (where appropriate) and (b) an effect estimate and its precision (e.g. confidence/credible interval), ideally using structured tables or plots.	8
Results of syntheses	20a	For each synthesis, briefly summarise the characteristics and risk of bias among contributing studies.	8 - 10
	20b	Present results of all statistical syntheses conducted. If meta-analysis was done, present for each the summary estimate and its precision (e.g. confidence/credible interval) and measures of statistical heterogeneity. If comparing groups, describe the direction of the effect.	8 - 10
	20c	Present results of all investigations of possible causes of heterogeneity among study results.	8 - 10
	20d	Present results of all sensitivity analyses conducted to assess the robustness of the synthesized results.	8 - 10
Reporting biases	21	Present assessments of risk of bias due to missing results (arising from reporting biases) for each synthesis assessed.	8 - 10
Certainty of evidence	22	Present assessments of certainty (or confidence) in the body of evidence for each outcome assessed.	8 - 10
**DISCUSSION**			
Discussion	23a	Provide a general interpretation of the results in the context of other evidence.	10 - 12
	23b	Discuss any limitations of the evidence included in the review.	12, 13
	23c	Discuss any limitations of the review processes used.	12, 13
	23d	Discuss implications of the results for practice, policy, and future research.	11 - 13
**OTHER INFORMATION**			
Registration and protocol	24a	Provide registration information for the review, including register name and registration number, or state that the review was not registered.	5
	24b	Indicate where the review protocol can be accessed, or state that a protocol was not prepared.	5
	24c	Describe and explain any amendments to information provided at registration or in the protocol.	NA

Section and Topic	Item #	Checklist item	Location where item is reported
Support	25	Describe sources of financial or non-financial support for the review, and the role of the funders or sponsors in the review.	14, 15
Competing interests	26	Declare any competing interests of review authors.	14, 15
Availability of data, code and other materials	27	Report which of the following are publicly available and where they can be found: template data collection forms; data extracted from included studies; data used for all analyses; analytic code; any other materials used in the review.	NA

From: Moher D, Liberati A, Tetzlaff J, Altman DG, The PRISMA Group (2009). Preferred Reporting Items for Systematic Reviews and Meta-Analyses: The PRISMA Statement. PLoS Med 6(6): e1000097. doi:10.1371/journal.pmed1000097.
PRISMA-P: Preferred Reporting Items for Systematic Reviews and Meta-Analyses-Protocols.

Table S2. Definition of moderate or severe AECOPD as reported in the RCTs included in the network meta-analysis.

Study	Definition of AECOPD
Rabe et al., 2020, ETHOS [15,21]	A moderate AECOPD led to treatment with antibiotics or systemic glucocorticoids, or both for at least 3 days. A severe AECOPD resulted in hospitalization or death.
Ferguson et al., 2018, KRONOS, [17]	Change in the patient's usual COPD symptoms that lasted ≥2 days, beyond normal day-to-day variation, acute in onset, and leading to a change in regular medication, including at least one major symptom (dyspnoea, sputum volume, or sputum colour) and at least one other major or minor symptom (cough, wheeze, sore throat, cold symptoms [rhinorrhoea or nasal congestion], or fever without other cause). AECOPD was categorised as: moderate (led to treatment with systemic corticosteroids, antibiotics, or both for ≥3 days, or ≥1 depot injectable dose of corticosteroids); or severe (led to hospital admission or a visit to a healthcare facility, e.g. emergency department, that lasted ≥24 hours, or COPD-related death).
Lipson et al., 2018, IMPACT [16,22]	A moderate AECOPD led to treatment with antibiotics or systemic glucocorticoids. A severe AECOPD resulted in hospitalization or death.
Singh et al., 2016, TRILOGY [18]	Worsening of the patient's respiratory symptoms that in the view of the patient's health-care provider required treatment with systemic corticosteroids, antibiotics, or hospital admission, or a combination of these. Events were classified as moderate or severe according to European Medicines Agency/Committee for Medicinal Products for Human Use guidelines (available at http://www.ema.europa.eu/docs/en_GB/document_library/Scientific_guideline/2012/08/WC500130880.pdf), with severe AECOPD being those requiring hospital admission or resulting in death.

AECOPD: acute exacerbation of COPD; COPD: chronic obstructive pulmonary disease; RCT: randomized controlled trial.

Supplementary Figures

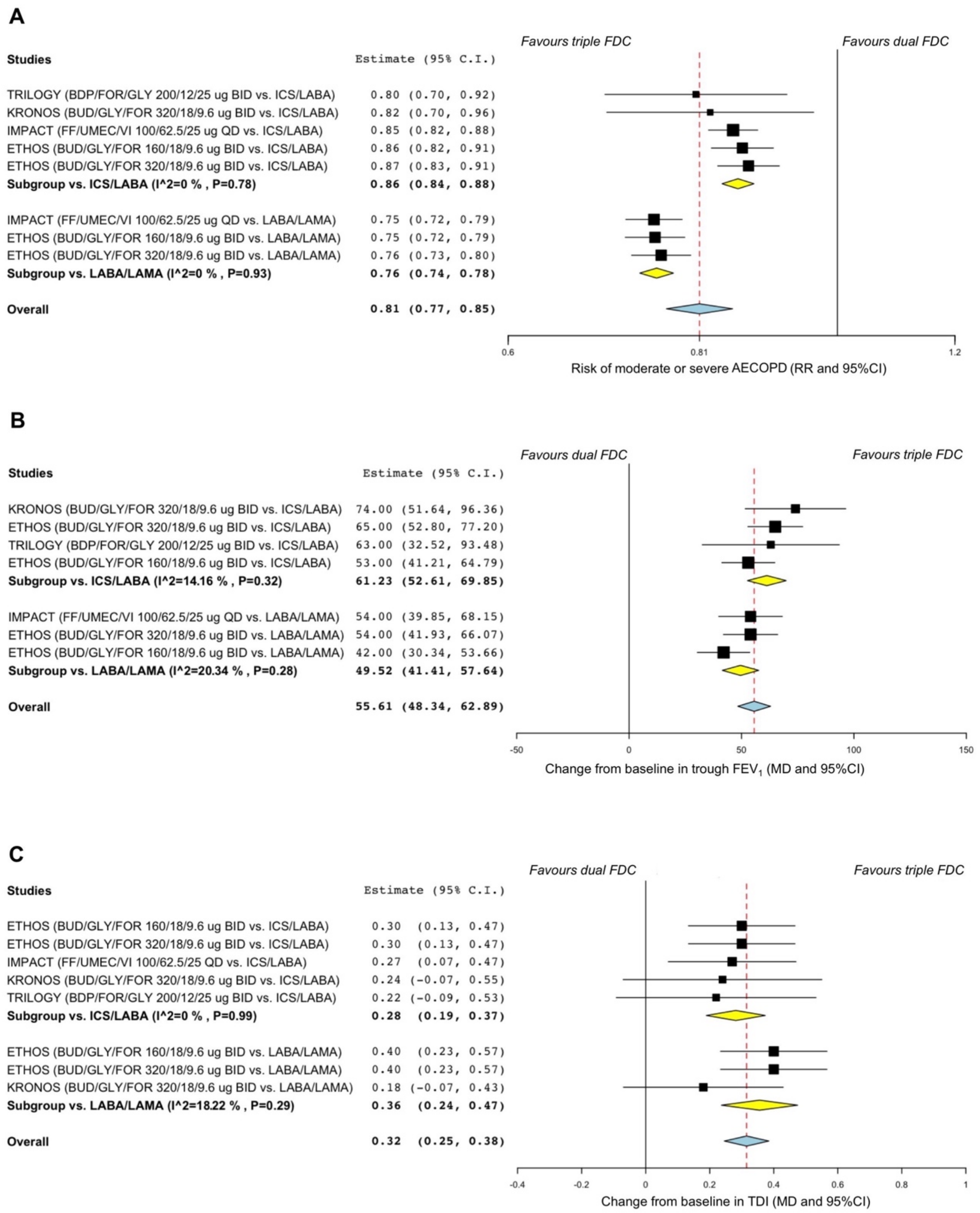

Figure S1. Forest plots of the sensitivity analysis performed by excluding the comparisons that introduced substantial heterogeneity in the overall pairwise meta-analysis of efficacy profile. AECOPD: acute exacerbation of COPD; BDP: beclomethasone dipropionate; BID: bis in die, twice daily; BUD: budesonide; COPD: chronic obstructive pulmonary disease; FDC: fixed-dose combination; FEV_1: forced expiratory volume in the first second; FF: fluticasone furoate; FOR: formoterol fumarate; GLY: glycopyrronium bromide or glycopyrrolate; ICS: inhaled corticosteroid; LABA: long-acting β_2-adrenoceptor agonist; LAMA: long-acting muscarinic antagonist; MD: mean difference; QD: quaque die, once daily; RR: relative risk; TDI: transition dyspnoea index; UMEC: umeclidinium bromide; VI: vilanterol.

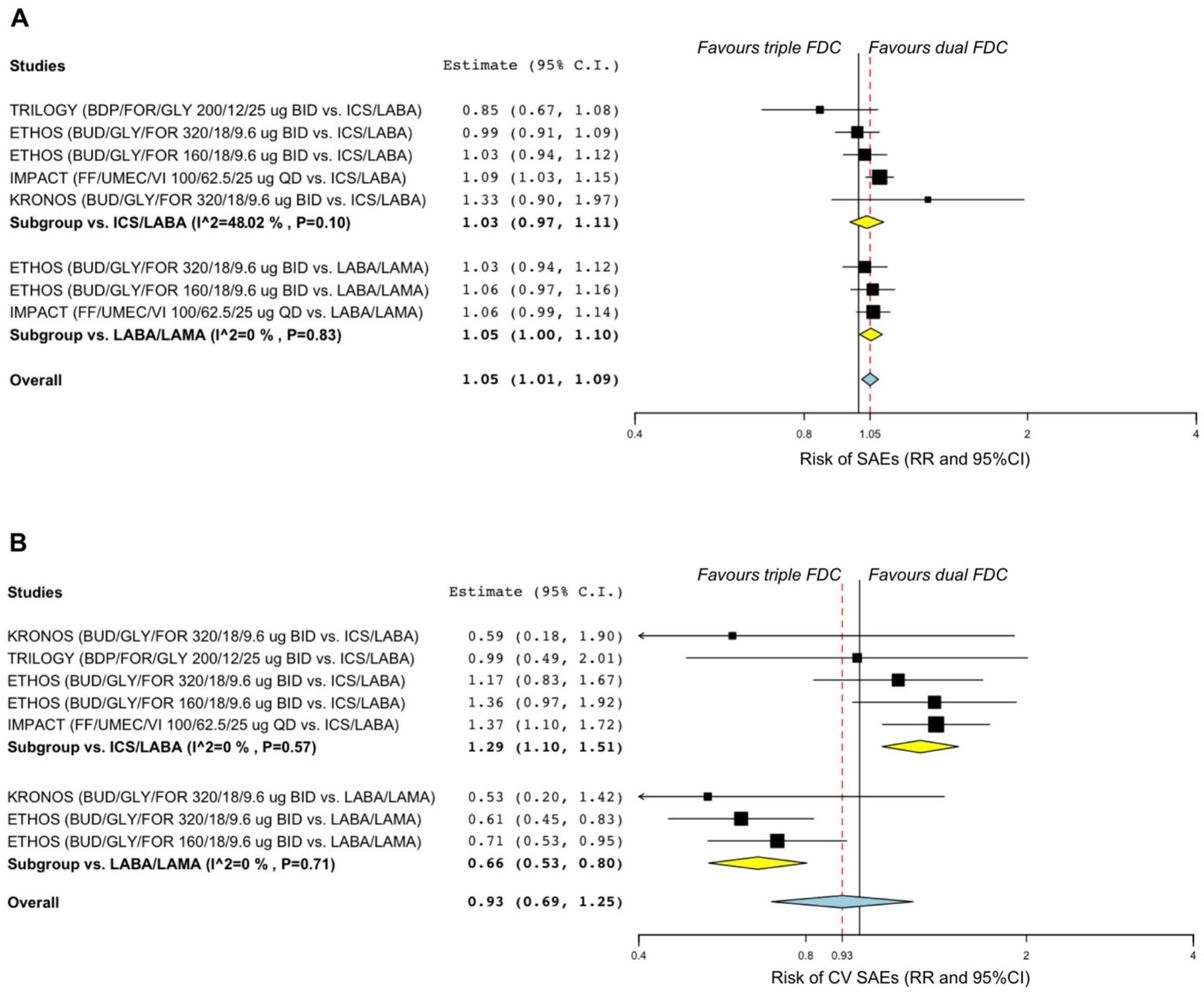

Figure S2. Forest plots of the sensitivity analysis performed by excluding the comparisons that introduced substantial heterogeneity in the overall pairwise meta-analysis of safety profile. BDP: beclomethasone dipropionate; BID: bis in die, twice daily; BUD: budesonide; CV: cardiovascular; FDC: fixed-dose combination; FF: fluticasone furoate; FOR: formoterol fumarate; GLY: glycopyrronium bromide or glycopyrrolate; ICS: inhaled corticosteroid; LABA: long-acting β_2-adrenoceptor agonist; LAMA: long-acting muscarinic antagonist; QD: quaque die, once daily; RR: relative risk; SAE: serious adverse event; UMEC: umeclidinium bromide; VI: vilanterol.

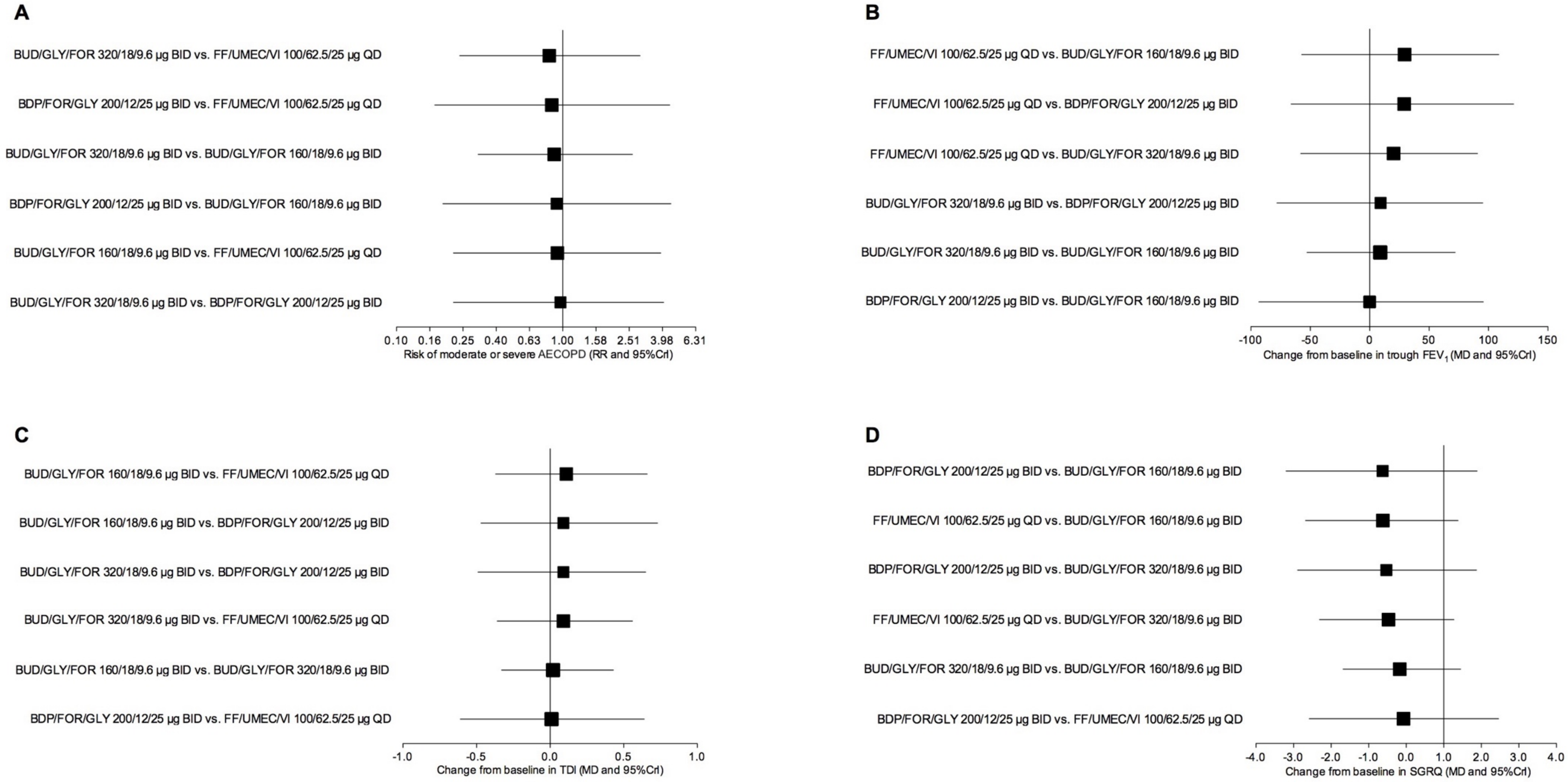

Figure S3. Forest plots of the overall network meta-analysis of efficacy profile. AECOPD: acute exacerbation of COPD; BDP: beclomethasone dipropionate; BID: bis in die, twice daily; BUD: budesonide; COPD: chronic obstructive pulmonary disease; CV: cardiovascular; FEV_1: forced expiratory volume in the first second; FF: fluticasone furoate; FOR: formoterol fumarate; GLY: glycopyrronium bromide or glycopyrrolate; MD: mean difference; QD: quaque die, once daily; RR: relative risk; SGRQ: St. George's Respiratory Questionnaire; TDI: transition dyspnea index; UMEC: umeclidinium bromide; VI: vilanterol; 95%CrI: 95% credible interval.

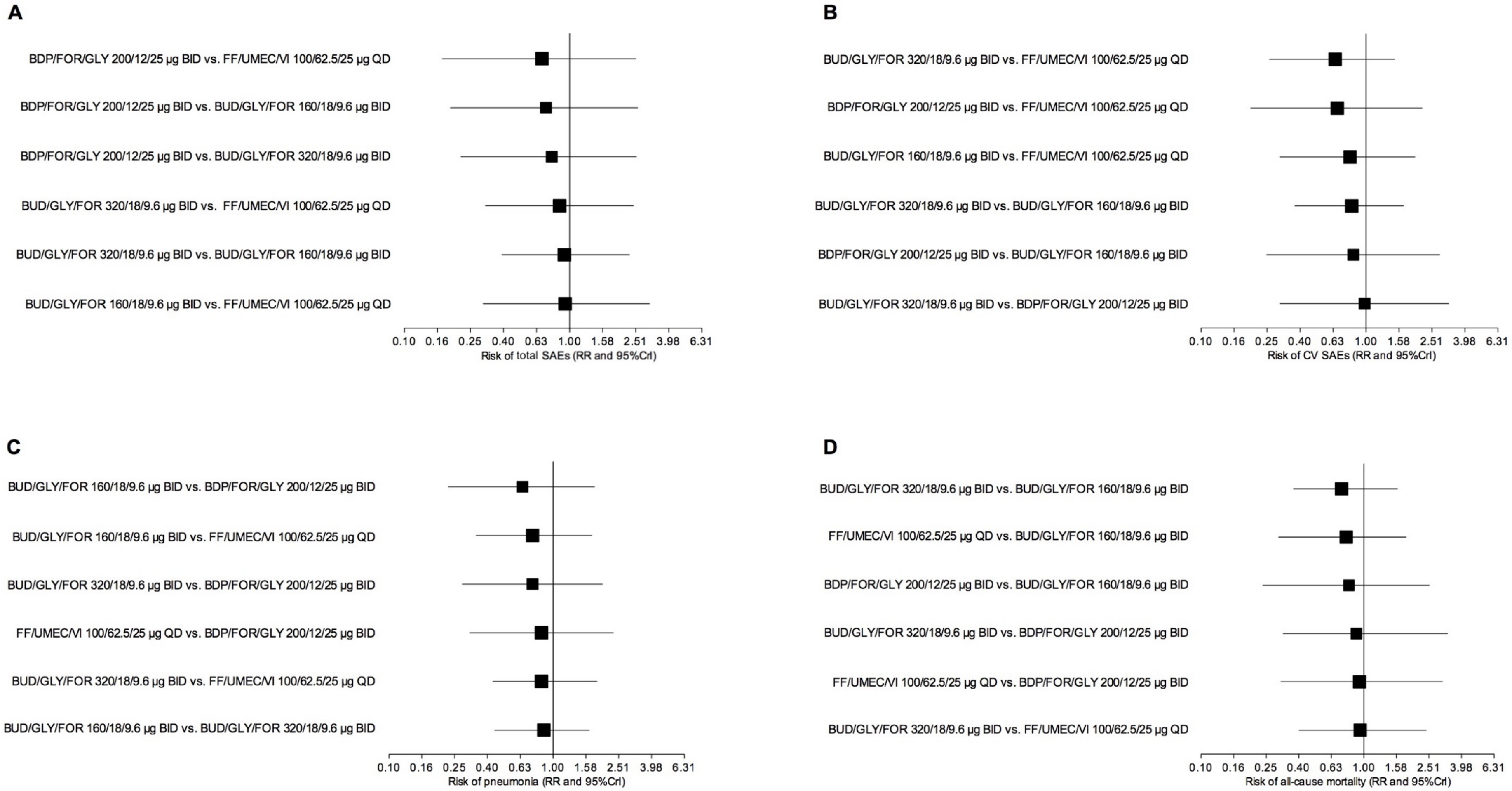

Figure S4. Forest plots of the overall network meta-analysis of safety profile. BDP: beclomethasone dipropionate; BID: bis in die, twice daily; BUD: budesonide; CV: cardiovascular; FEV_1: forced expiratory volume in the first second; FF: fluticasone furoate; FOR: formoterol fumarate; GLY: glycopyrronium bromide or glycopyrrolate; GRADE: QD: quaque die, once daily; RR: relative risk; SAE: serious adverse event; UMEC: umeclidinium bromide; VI: vilanterol; 95%CrI: 95% credible interval.

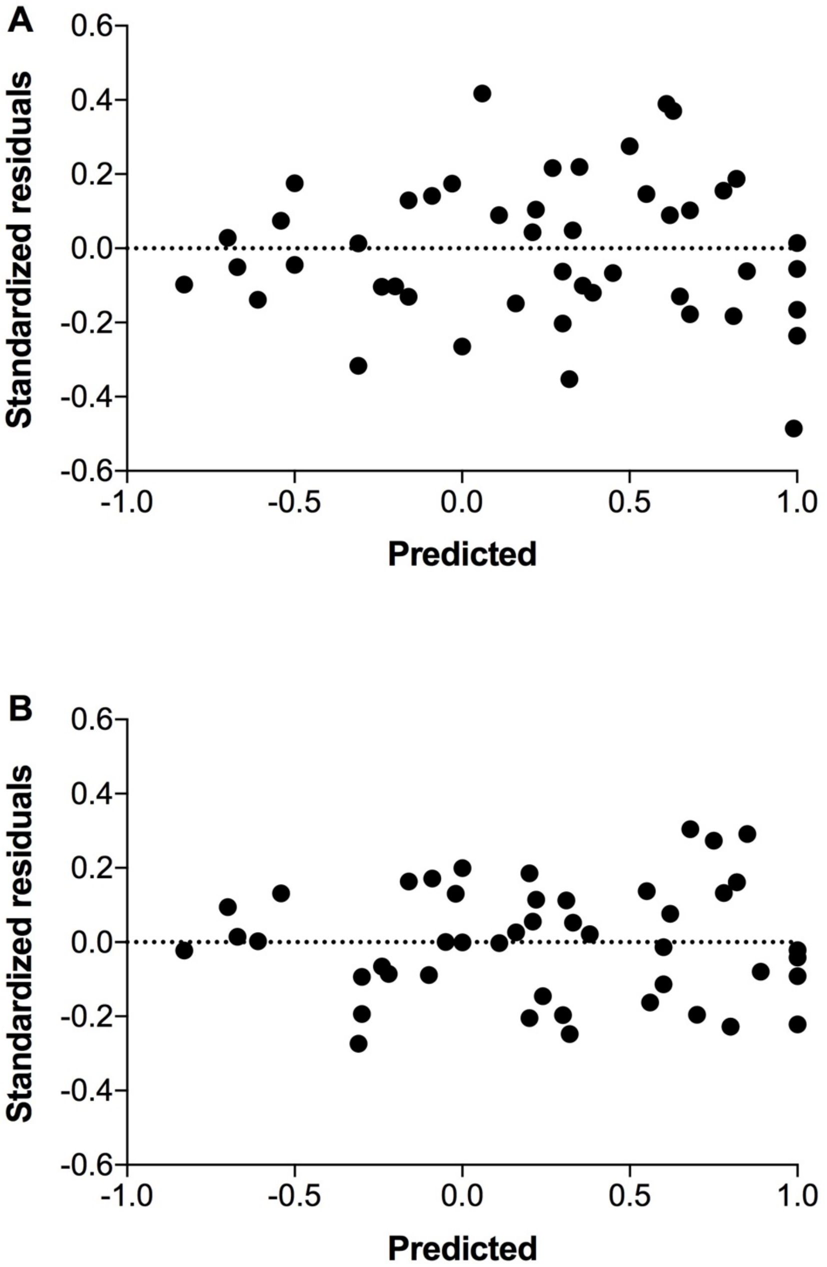

Figure S5. Residual plot of the overall consistency/inconsistency regression before (A) and after sensitivity analysis (B) to reduce the risk of bias in the overall Bayesian network.

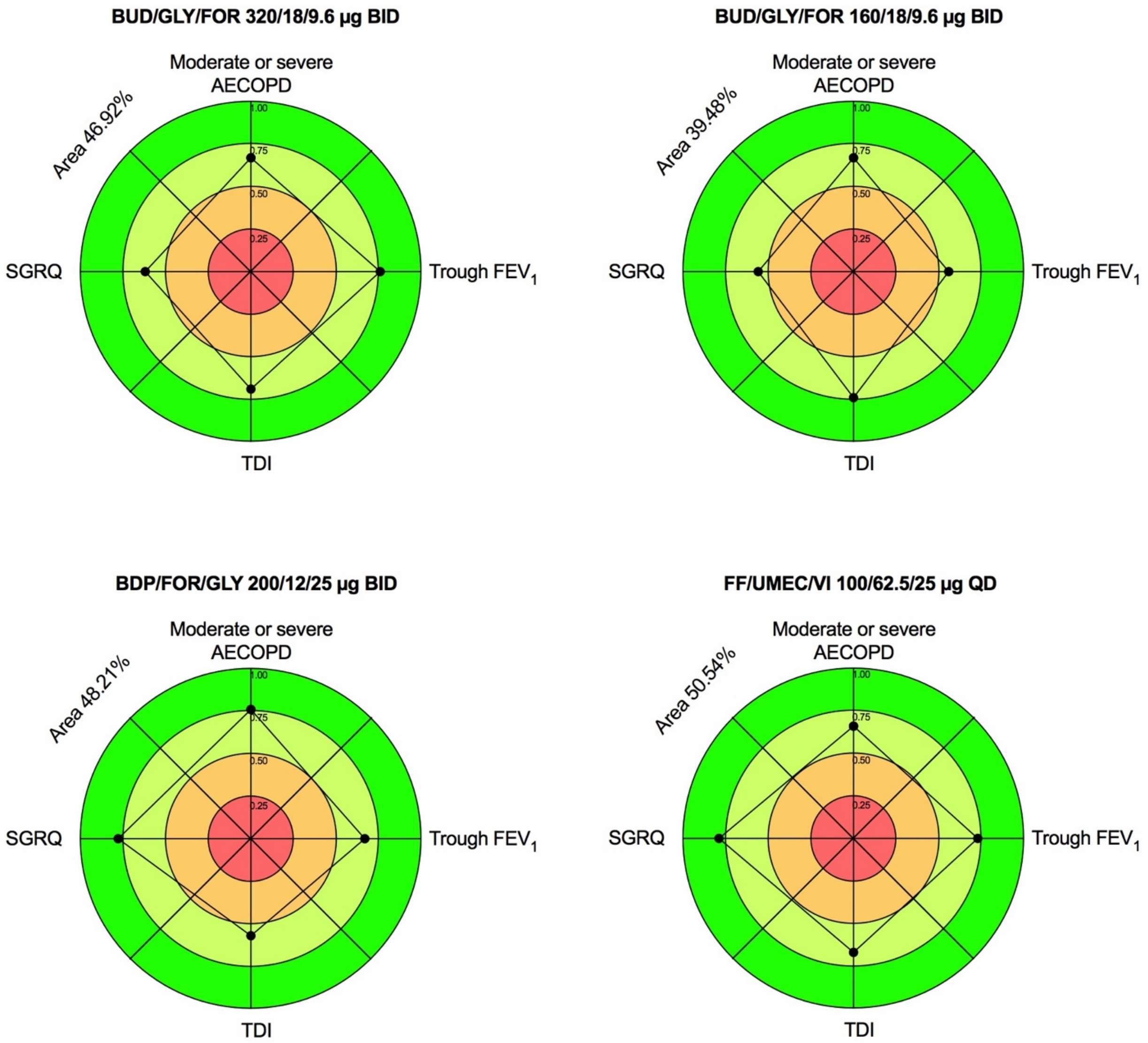

Figure S6. Graphical representation of efficacy profile of ICS/LABA/LAMA FDCs in COPD patients according the IBiS score: the greater the area, the better the efficacy profile. AECOPD: acute exacerbation of COPD; BDP: beclomethasone dipropionate; BID: bis in die, twice daily; BUD: budesonide; COPD: chronic obstructive pulmonary disease; FDC: fixed-dose combination; FEV_1: forced expiratory volume in the first second; FF: fluticasone furoate; FOR: formoterol fumarate; GLY: glycopyrronium bromide or glycopyrrolate; ICS: inhaled corticosteroid; IBiS: Implemented Bidimensional SUCRA; LABA: long-acting β_2-adrenoceptor agonist; LAMA: long-acting muscarinic antagonist; QD: quaque die, once daily; SGRQ: St. George Respiratory Questionnaire; SUCRA: surface under the cumulative ranking curve; TDI: transitional dyspnea index; UMEC: umeclidinium bromide; VI: vilanterol.

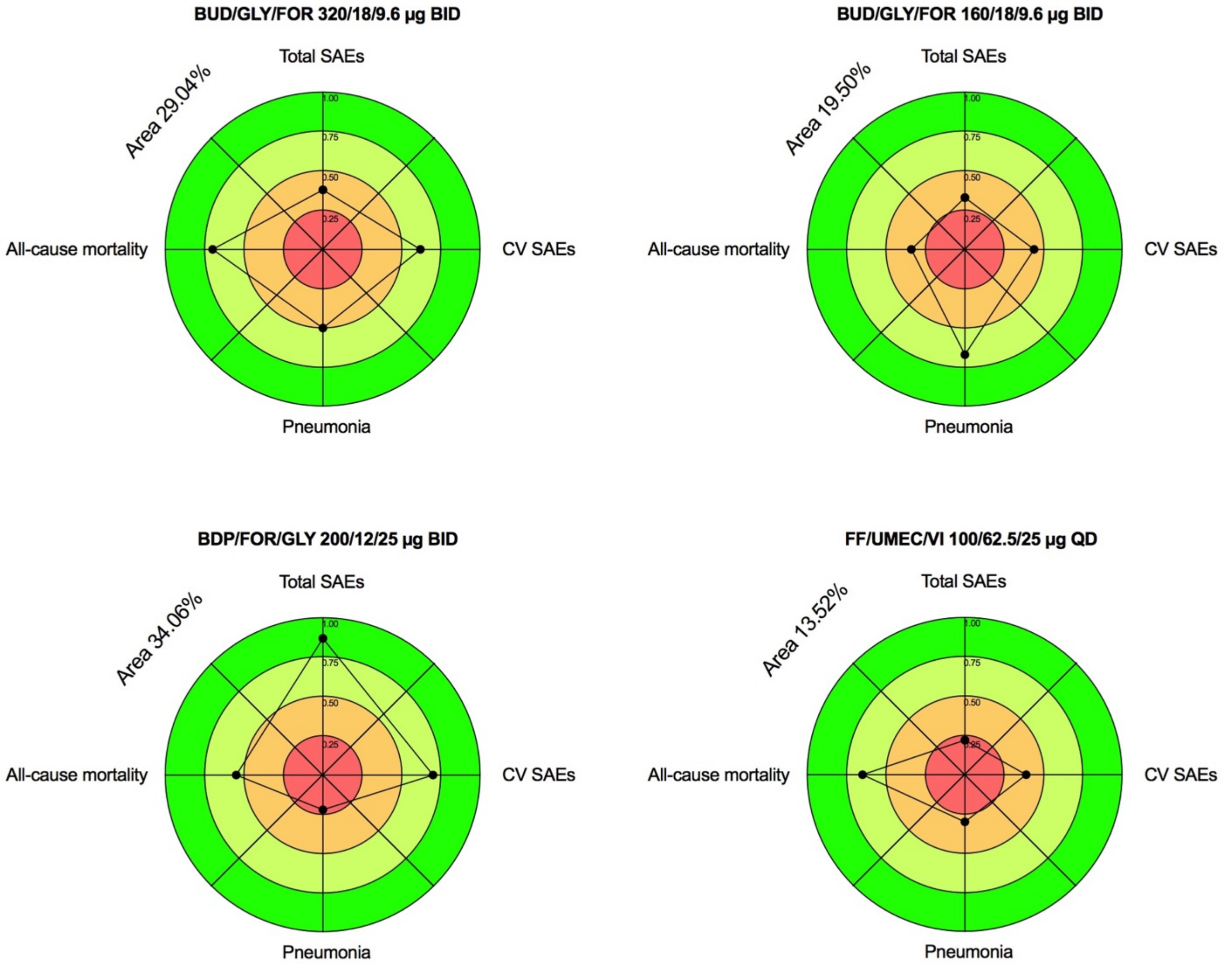

Figure S7. Graphical representation of safety profile of ICS/LABA/LAMA FDCs in COPD patients according the IBiS score: the greater the area, the better the safety profile. BDP: beclomethasone dipropionate; BID: bis in die, twice daily; BUD: budesonide; COPD chronic obstructive pulmonary disease; CV: cardiovascular; FDC: fixed-dose combination; FF: fluticasone furoate; FOR: formoterol fumarate; GLY: glycopyrronium bromide or glycopyrrolate; ICS: inhaled corticosteroid; IBiS: Implemented Bidimensional SUCRA; LABA: long-acting β_2-adrenoceptor agonist; LAMA: long-acting muscarinic antagonist; QD: quaque die, once daily; SAEs: serious adverse events; SUCRA: surface under the cumulative ranking curve; UMEC: umeclidinium bromide; VI: vilanterol.

A

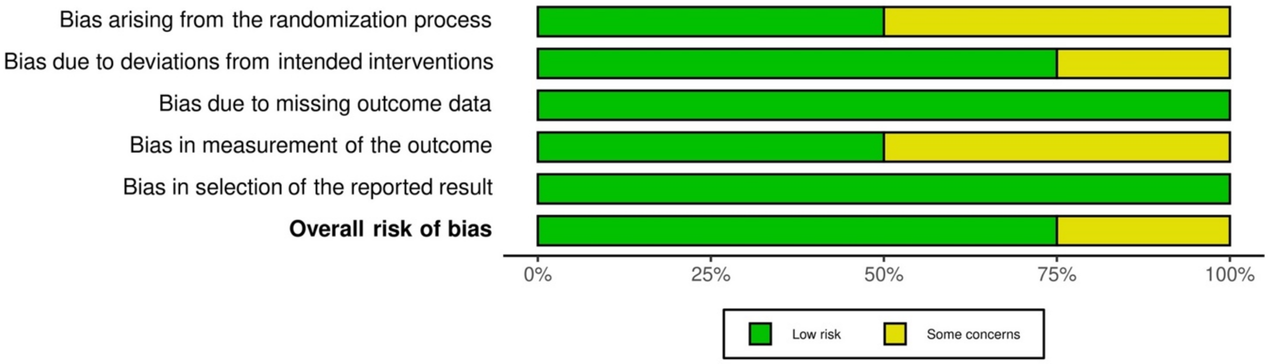

B

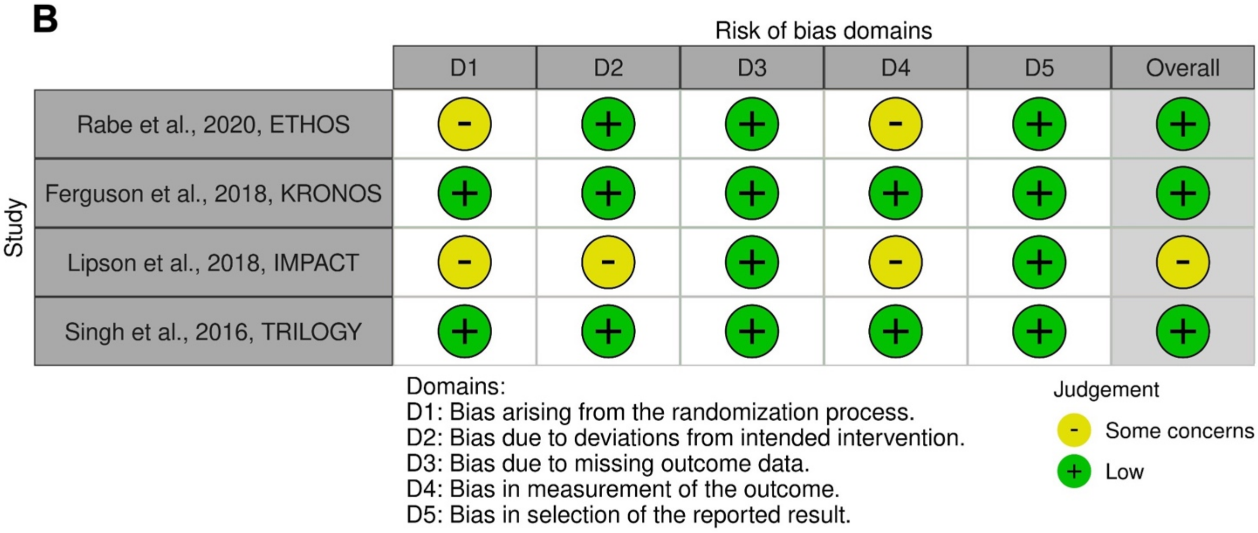

Figure S8. Assessment of the risk of bias via the weighted plot for the assessment of the overall risk of bias (A) and the traffic light plot of the risk of bias of each included RCT via the Cochrane RoB 2 tool (B) (n=4 studies). Traffic light plot reports five risk of bias domains: D1, bias arising from the randomization process; D2, bias due to deviations from intended intervention; D3, bias due to missing outcome data; D4, bias in measurement of the outcome; D5, bias in selection of the reported result; Yellow circle indicates some concerns on the risk of bias and green circle represents low risk of bias. RCT: randomized controlled trial; RoB: risk of bias.
